# The Combined Effect of Promoting the Mediterranean Diet and Physical Activity on Metabolic Risk Factors in Adults: A Systematic Review and Meta-Analysis of Randomised Controlled Trials

**DOI:** 10.3390/nu10111577

**Published:** 2018-10-25

**Authors:** Evangelia Malakou, Manolis Linardakis, Miranda Elaine Glynis Armstrong, Dimitra Zannidi, Charlie Foster, Laura Johnson, Angeliki Papadaki

**Affiliations:** 1 Centre for Exercise, Nutrition and Health Sciences, School for Policy Studies, University of Bristol, 8 Priory Road, Bristol BS8 1TZ, UK; em16938.2016@grouting.my.bristol.ac.uk (E.M.); miranda.armstrong@bristol.ac.uk (M.E.G.A.); charlie.foster@bristol.ac.uk (C.F.); laura.johnson@bristol.ac.uk (L.J.); 2 Department of Social Medicine, Faculty of Medicine, University of Crete, Heraklion 71003, Crete, Greece; linman@med.uoc.gr; 3 Department of Nutrition and Dietetics, School of Health Science and Education, Harokopio University, Eleftheriou Venizelou 70, Kallithea 176 76, Athens, Greece; dimitra.zannidi@gmail.com

**Keywords:** mediterranean diet, physical activity, metabolic risk factors, cardiovascular disease, randomised controlled trials, systematic review, meta-analysis

## Abstract

Adhering to the Mediterranean diet (MD) and physical activity (PA) public health guidelines have independently been linked to health benefits in adults. These behaviours form essential components of the traditional Mediterranean lifestyle. However, their combined effect on metabolic risk has not been systematically assessed. This systematic review with meta-analysis (PROSPERO; CRD42017073958) aimed to examine, for the first time, the combined effect of promoting the MD and PA compared with no treatment, treatment with MD or PA alone, or a different dietary and/or PA treatment, and estimate its magnitude on metabolic risk factors. Medline, Embase, CINAHL and Web of Science were systematically searched until March 2018 for English language controlled interventions reporting the combined effects of the MD and PA on one or multiple metabolic risk factors in adults. Two researchers independently conducted data extraction and risk of bias assessment using a rigorous methodology. Reporting followed PRISMA guidelines. Quality of reporting and risk of bias were assessed using the CONSORT guidelines and the Cochrane Collaboration’s tool, respectively. Data from 12 articles reporting 11 randomised controlled trials (*n* = 1684) were included in the qualitative synthesis; across them, risk of bias was considered low, unclear and high for 42%, 25% and 33% of domains, respectively. Between-study heterogeneity ranged from 44% (triglycerides) to 98% (insulin and high density lipoprotein cholesterol (HDL)-cholesterol). Compared to a control condition, there was strong evidence (*p* < 0.001) of a beneficial effect of promoting the MD and PA on body weight (−3.68 kg, 95% CI (confidence intervals) −5.48, −1.89), body mass index (−0.64 kg/m^2^, 95% CI −1.10, −0.18), waist circumference (−1.62 cm, 95% CI −2.58, −0.66), systolic (−0.83 mmHg, 95% CI −1.57, −0.09) and diastolic blood pressure (−1.96 mmHg, 95% CI −2.57, −1.35), HOMA-IR index (−0.90, 95% CI −1.22, −0.58), blood glucose (−7.32 mg/dL, 95% CI −9.82, −4.82), triglycerides (−18.47 mg/dL, 95% CI −20.13, −16.80), total cholesterol (−6.30 mg/dL, 95% CI −9.59, −3.02) and HDL-cholesterol (+3.99 mg/dL, 95% CI 1.22, 6.77). There was no evidence of an effect on insulin concentrations. The data presented here provide systematically identified evidence that concurrently promoting the MD and PA is likely to provide an opportunity for metabolic risk reduction. However, due to the high degree of heterogeneity, most likely due to the variation in control group treatment, and the small number of included studies, findings from the pooled analysis should be interpreted with caution. These findings also highlight the need for high quality randomised controlled trials examining the combined effect of the MD and PA on metabolic risk.

## 1. Introduction

The traditional Mediterranean diet (MD) has been consistently associated with reduced risk of non-communicable diseases (NCD), including cardiovascular disease (CVD) [1], the metabolic syndrome [2] and type 2 diabetes [3]. Recent meta-analyses of randomised controlled trials (RCTs) have also shown that promoting adherence to the MD improves several metabolic risk factors, including waist circumference, blood pressure, triglyceride and glucose levels [1,4], as well as body weight [1,5], total and high-density lipoprotein (HDL) cholesterol and glycosylated haemoglobin (HbA1c) [1]. Characterised by high intake of olive oil and plant foods (fruits, vegetables, legumes, nuts and non-refined cereals), low-to-moderate intake of dairy products, fish and poultry, moderate intake of alcohol and low intake of red meat and sweets, the MD has been recognised as a model for healthy eating that should be promoted not only to Mediterranean countries, but also to populations outside of the Mediterranean region [6].

Regular physical activity (PA) was an integral part of the traditional Mediterranean lifestyle [6,7]. Systematic reviews of prospective cohort studies have found that high, compared to low, levels of PA are associated with reduced risk of CVD [8], type 2 diabetes [9,10,11] and the metabolic syndrome [12]. PA has also been shown to result in improvements in metabolic risk factors, such as blood pressure [13,14], HbA1c [13,15], homeostatic model assessment-insulin resistance (HOMA-IR) [16], adiposity [13,17], triglycerides [16] and HDL-cholesterol [16,18], particularly in individuals at high CVD risk or those with high levels of these markers at baseline. However, results often vary according to different types (e.g., endurance, resistance, aerobic, etc.), duration and intensity or volume of PA [19]. The World Health Organisation recommends performing moderate-intensity PA for ≥150 min/week and muscle strengthening PA for ≥2 days/week [20] for adults to have these health benefits.

Promoting the PA guidelines in the context of the traditional Mediterranean lifestyle [6,7] provides a more comprehensive endorsement of a lifestyle that has been associated with longevity and reduced risk of NCDs beyond the MD alone [21]. A recent US cohort study suggested that adhering to recommended PA levels and the MD guidelines is associated with lower risk of overall mortality (RR (risk ratio) = 0.82; 95% CI (confidence intervals) 0.79, 0.85) than only adhering to the MD (RR = 0.86; 95% CI 0.83, 0.88) or PA guidelines (RR = 0.86; 95% CI 0.84, 0.89) [22]. This was supported by a cohort study in Spain, where high MD adherence and engaging in moderate/high levels of PA (versus low) was associated with a lower likelihood of all-cause mortality (HR (hazard ratio) = 0.36; 95% CI 0.19, 0.67), compared to high MD adherence (HR = 0.66; 95% CI 0.46, 0.96) or high PA (HR = 0.48; 95% CI 0.33, 0.71) alone [23]. In the same cohort, it was suggested that additional benefits of a combination of high MD adherence and a highly active lifestyle were extended to CVD incidence (HR = 0.25; 95% CI 0.13, 0.48), larger than those obtained from high MD adherence (HR = 0.33; 95% CI 0.20, 0.55) or engaging in high levels of PA (HR = 0.43; 95% CI 0.20, 0.90) alone [24]. Thus, it is viable to hypothesise that promoting the MD and PA public health guidelines together might provide an opportunity to obtain greater health benefits, over and above those acquired separately by the MD and PA. Nevertheless, well-designed, controlled interventions are needed to confirm the combined effect of the MD and PA on metabolic risk and subsequent disease outcomes.

To date, only one meta-analysis of RCTs has reported on the combined effect of promoting the MD and PA on body weight, indicating greater weight loss when interventions combined advice on the MD and PA (−4.01 kg; 95% CI −5.79, −2.23 kg), compared to the MD alone (−1.75 kg; 95% CI, −2.86, −0.64 kg) [5]. This finding was the result of a sensitivity analysis, however, as the study’s primary aim was to examine the effect of the MD, instead of the combined effect of MD and PA, on body weight. Further, this meta-analysis did not report on other metabolic risk factors [5], which is important to establish the effect of promoting both behaviours on overall metabolic health. To answer the research question “what is the combined effect of the MD and PA on metabolic risk?”, the aim of this study was therefore to conduct, for the first time, a systematic review, with meta-analysis, of controlled trials assessing the combined effect of promoting the MD and PA on metabolic risk in adults. The specific objectives were to: (1) identify and review controlled trials assessing the combined effect of the MD and PA, compared with no treatment, treatment with MD or PA alone, or a different dietary and/or PA treatment, on metabolic risk factors in adults; and (2) calculate the magnitude of effect of the intervention on metabolic risk factors.

## 2. Methods

The review protocol was registered with the International Prospective Register of Systematic Reviews (PROSPERO; CRD42017073958) and reported according to the Preferred Reporting Items for Systematic reviews and Meta-Analyses (PRISMA) statement (http://www.prisma-statement.org/PRISMAStatement/) [25,26] (Supplementary Materials I, Table S1).

### 2.1. Study Eligibility Criteria

#### 2.1.1. Participants

Studies were included in the review if they involved healthy or unhealthy adults (aged ≥18 years), including those with established metabolic diseases (e.g., type 2 diabetes, hypertension, CVD, etc.). Studies conducted in children and/or adolescents were excluded.

#### 2.1.2. Study Design

All controlled trials (randomised or non-randomised), reporting outcome measures at pre- and post-intervention and of any length of follow-up, were included. Studies with other methodological designs (e.g., cohort, case-control, cross-sectional, etc.), and study protocols and conference abstracts were excluded.

#### 2.1.3. Intervention(s)

Interventions promoting a combination of MD and PA were included. Studies using any type of intervention delivery and duration and promoting any type of PA or exercise training were included. Studies were only included if they promoted the whole MD or Mediterranean-style dietary pattern, and excluded if they promoted single components of the MD (e.g., fruits, nuts, etc.). Studies were also excluded if the intervention contained components of other lifestyle behaviours, such as stress management or smoking cessation, as these might have potentially prevented our ability to isolate the effect to the combination of MD and PA alone.

#### 2.1.4. Comparator(s)

Studies were included if the comparator/control group received no treatment, or one of the intervention components alone (i.e., MD only or PA only). Studies were also included if the control group received advice to follow a different dietary pattern or diet (e.g., a low-fat diet) and/or PA treatment (e.g., different in volume and intensity compared to the intervention group). Studies were excluded if they did not have a comparison group, or if both the intervention and control groups received a combined treatment of MD and PA.

#### 2.1.5. Outcome Measures

Studies were included if they involved a measure of one or more metabolic risk factors (body weight (BW), body mass index (BMI), waist circumference (WC), systolic (SBP) and/or diastolic (DBP) blood pressure, and blood levels of glucose, insulin, HbA1c, HOMA-IR, total cholesterol, LDL- and HDL-cholesterol and triglycerides (TG)), either as a primary or secondary outcome.

### 2.2. Search Strategy

Original studies that were published in English up to 27 March 2018 were included in this review. The systematic search was developed by three researchers (E.M., M.E.G.A. and A.P.) and conducted (by E.M.) in Medline (OvidSP), Embase, Web of Science-Core Collection and CINAHL (Cumulative Index to Nursing & Allied Health Literature) using terms related to the MD, PA or exercise, metabolic risk factors and controlled trials. The detailed search strategy and terms for each database can be found in Supplementary Materials II. Reference lists of the included articles and relevant systematic reviews and/or meta-analyses were also searched for additional references.

### 2.3. Study Selection

After the removal of duplicates, all articles identified through the database searches were assessed for relevance by one reviewer (E.M.) and 10% were also assessed by a second independent reviewer (D.Z.), using information contained in the title and abstract. If there were doubts on whether a study should be included from screening the title and abstract, its full text was accessed to establish eligibility. The full text of all eligible articles was then obtained and read by one reviewer (E.M.), and checked by either A.P. or M.E.G.A. to ensure articles met the inclusion and exclusion criteria of the review. Corresponding authors were also contacted if clarification on any aspect of the eligible studies was required. At every stage of study selection, any uncertainties were resolved by discussion (between E.M., D.Z., M.E.G.A. and A.P.) until consensus was reached.

### 2.4. Data Extraction

Data extraction was conducted using the Cochrane data collection form for reviews of RCTs and non-RCTs (available at http://training.cochrane.org/resource/data-collection-forms-intervention-reviews). The form included six parts for which data were extracted: (i) general information (e.g., publication type); (ii) study eligibility (e.g., type of study, participants, type of intervention and comparator); (iii) characteristics of included studies (methods, participants, intervention and control groups, outcomes, and other characteristics); (iv) risk of bias assessment; (v) data and analysis; and (vi) other information. When records of the same study but reporting on different time periods (e.g., different follow-ups) existed, data extraction was conducted individually for each record (to assess potential long-term effects of the intervention) and then all information was combined in the same data extraction form. If multiple articles of the same study reported on the study details or same outcomes, those manuscripts were combined in a single data extraction form, with the article reporting the most complete data for the purposes of the current review being considered the primary source. Data were extracted by two independent reviewers (E.M. and A.P.) and any disagreements were resolved by discussion with a third reviewer (M.E.G.A.).

### 2.5. Quality Assessment

The reporting quality of included RCTs was assessed according to the CONSORT guidelines [27]. The CONSORT checklist (available at http://www.consort-statement.org/) was used to explore to what extent each included article reported information in the sections of title and abstract, introduction, methods, results and discussion, according to standards for reporting [27,28]. If an included article was not an RCT, the 22-item ΤREND statement was to be used to assess the quality of reporting [29].

### 2.6. Risk of Bias

For each included article, risk of bias was independently assessed by two reviewers (E.M. and A.P.) using the Cochrane Collaboration’s tool for assessing risk of bias in randomised trials [30]. Disagreements were resolved by discussion with a third reviewer (M.E.G.A.). Random sequence allocation, allocation concealment, blinding of participants and personnel, blinding of outcome assessment, incomplete outcome data, selective outcome reporting and other sources of potential bias (such as compliance to the treatment) were items assessed as having “low”, “high” or “unclear” risk, based on standard criteria described in the Cochrane handbook [31]. When included articles reported that procedures had been conducted with appropriate methods, the relevant items were assessed as “low risk”. When articles reported methods likely to introduce bias, the relevant items were assessed as “high risk”, whereas when sufficient information was not provided to reach a decision, the relevant items were assessed as “unclear risk” [31]. As the use of scales to calculate a summary risk of bias score for each study is discouraged by the Cochrane handbook [31], risk of bias was summarised in a figure: (i) for each included article and every item; and (ii) as a percentage of articles assessed as having low, high and unclear risk of bias for each item in the Cochrane Collaboration’s tool.

### 2.7. Statistical Analyses

Inter-rater reliability was calculated using the Cohen’s kappa co-efficient (kappa = 0.69, *p* < 0.001, 92.6% agreement). For each included article, effect sizes were summarised for each outcome (metabolic risk factor) by calculating the mean difference between the intervention and control condition from the pre-intervention to the post-intervention period. Data on outcomes were transformed, where necessary, into the same measurement units using standard conversion factors, to enable between-study comparisons. If an included article reported multiple outcomes of interest to the current review, each outcome was evaluated and reported independently. If an included study reported multiple follow-ups for the same outcomes, findings from all follow-ups were evaluated for the qualitative review, but only the follow-up with the most complete data was considered for the meta-analysis.

A random-effects meta-analysis was used to summarise intervention effect estimates, expressed as mean differences (inverse variance) with 95% CIs. A fixed-effects model was used for triglyceride levels, as heterogeneity between studies assessing this outcome was lower, compared to other outcomes. Between-study heterogeneity was assessed using the *I*^2^ statistic, with values >50% indicating substantial heterogeneity [32]. Studies reporting outcomes as median (range) and for which an appropriate combination of means, standard deviations, standard errors or confidence intervals for the outcomes of interest was not available, were not included in the meta-analysis (details for these papers are shown in Table 1). Where appropriate, additional a posteriori defined subgroup analyses were conducted to explore whether intervention duration (≤2 years vs. >2 years) was a potential source of heterogeneity for the treatment effect. The meta-analysis was conducted using Review Manager (RevMan), Version 5.2 (The Nordic Cochrane Centre, The Cochrane Collaboration, Copenhagen, Denmark, 2012). Egger’s tests were not appropriate to conduct in order to assess publication bias, as fewer than 10 studies were included in the meta-analysis of each metabolic risk factor [33]. Funnel plots, created in RevMan, were used for visual evaluation of publication bias.

## 3. Results

### 3.1. Study Selection

**Figure 1:**
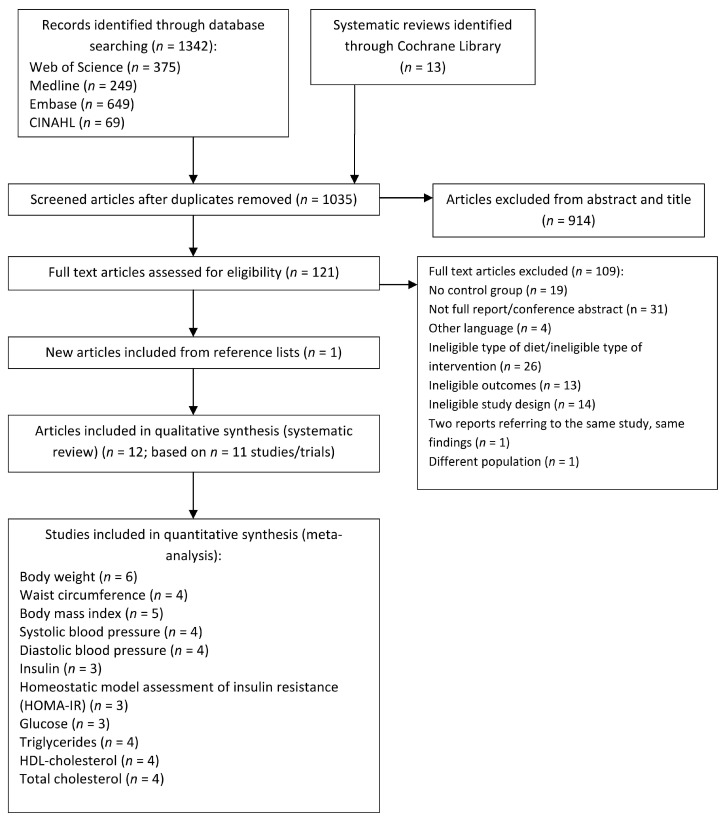
PRISMA flow diagram of literature search and study selection.

Figure 1 illustrates the literature search and study selection process [25,26]. Of 1035 unique records, 121 full-text articles were assessed for eligibility and 12 articles (representing 11 unique trials) were included in the qualitative systematic review [34,35,36,37,38,39,40,41,42,43,44,45] (the list of excluded articles can be found in Supplementary Materials I, Table S2). Two articles reported separately on the post-intervention (four years) [37] and extended follow-up (six years) [38] of the same study, therefore they were considered independently for the qualitative review but only the article with the most complete data [37] was included in the meta-analysis. One trial reported the outcomes of interest in two separate publications [43,46], which were merged together for the purposes of this review. Of the 11 included articles, the ones reporting sufficient data to be included in the meta-analysis ranged from three (for glucose, insulin and HOMA-R index) [37,39,40] to six (for body weight) [36,37,39,40,41,45] (Figure 1).

### 3.2. Study Characteristics

Table 1 summarises the characteristics of the included articles. All 11 studies were RCTs published since 2003. Eight studies were conducted in the Mediterranean region (Italy, Spain, Greece and Croatia), while remaining studies were carried out in Luxembourg [34], Germany [42] and Australia [35]. Sample sizes ranged from 30 [35] to 406 [41]. Of the total of 1684 participants in the included articles (mean age 47.6 ± 10.8 years), 951 (56.5%) were females, with one study recruiting only men [36] and three recruiting only women [35,40,42]. Most studies (*n* = 10) recruited participants within a clinical/health setting while one involved a university population [35]. All studies included participants with at least one metabolic disease, such as overweight/obesity, metabolic syndrome or type 2 diabetes. None of the studies focused on adults free of chronic disease risk factors. Between group changes from pre- to post-intervention were reported for body weight in 11 articles [34,35,36,37,38,39,40,41,43,44,45], WC in 7 [35,37,38,39,41,43,45], BMI in 9 [34,35,37,39,40,41,42,43,45], SBP and DBP in 7 [35,37,38,39,40,41,43], insulin in 6 [35,36,37,39,40,46], HOMA-IR in 5 [35,37,39,40,46], glucose in 7 [35,36,37,38,39,40,43], triglycerides and HDL-cholesterol in 8 [34,35,37,38,39,40,41,43], LDL-cholesterol in 4 [34,35,41,43] and total cholesterol in 9 [34,35,36,37,38,39,40,41,43] articles.

Intervention duration varied from twelve weeks (three months) [35,43] to six years [38] (Table 1). Of the participants in the included articles, 869 were assigned to a MD and PA intervention and 815 to a control condition (*n* = 70 receiving no treatment and *n* = 745 receiving a different treatment to the intervention). The dietary component of the intervention received by the intervention groups comprised of education on: the MD in two studies [34,44]; a low-energy MD in six studies [36,39,40,41,43,45] and a low-energy MD (if required) in one study [42]; a low-energy, low-carbohydrate MD in two studies [37,39]; and a low-glycaemic index MD in one study [35]. The PA component of the intervention in the intervention groups comprised of advice to increase PA in eight studies [34,36,37,38,39,40,41,44,45]; supervised exercise sessions in one study [35]; advice to increase PA and supervised sessions in one study [43]; and provision of individualised training plans in one study [42] (Table 1).

The control groups were asked to not change their usual dietary and PA habits in two studies [34,35]; and received advice to change only their dietary habits in two studies (general advice on healthy eating [36] and on a low-energy MD diet [43]). In the remaining studies, the control groups received advice on both diet and PA. This involved general advice on healthy eating and PA in three studies [40,41,42]; and advice to increase PA and follow a specific diet in four studies (low-energy, low-fat diet [37,38]; prudent diet [39]; low-fat diet [44]; and low-energy prudent diet [45]) (Table 1).

Studies varied widely with regards to intervention mode and intensity of delivery. Compliance to the MD and PA components of the intervention was assessed via self-reported methods, such as dietary and PA questionnaires and/or diaries, in all studies. One study [43] did not report the method of assessing compliance to PA and one study further assessed compliance by attendance of participants to supervised training sessions [42].

### 3.3. Effect on Metabolic Risk Factors

Table S3 (Supplementary Materials I) presents the summary of the findings (between-group differences) for all 12 reports of the 11 studies included in the systematic review. For the articles which reported the metabolic risk factors of interest, the combined effect of the MD and PA, as compared with a control condition, showed a protective effect for BW in 5/11 articles, for WC in 4/7 articles, for BMI in 4/9 articles, for SBP in 4/7 articles, for DBP in 4/7 articles, for glucose levels in 4/7 articles, for insulin in 5/6 articles, for HOMA-IR index in 4/5 articles, for total cholesterol in 2/9 articles, for LDL-cholesterol in 1/4 articles, for HDL-cholesterol in 5/8 articles and for triglyceride levels in 6/8 articles.

In the pooled analysis (Table 2 and Supplementary Materials I, Figures S1–S11), there was strong evidence (*p* < 0.001) of a greater beneficial effect of the combination of MD and PA, compared to a control condition, on 10 of the 11 metabolic risk factors of interest. Effect estimates suggested that the combined effect of the MD and PA resulted in greater decreases in BW (−3.68 kg, 95% CI −5.48, −1.89), WC (−1.62 cm, 95% CI −2.58, −0.66), BMI (−0.64 kg/m^2^, 95% CI −1.10, −0.18), SBP (−0.83 mmHg, 95% CI −1.57, −0.09), DBP (−1.96 mmHg, 95% CI −2.57, −1.35), HOMA-IR index (−0.90, 95% CI −1.22, −0.58), glucose levels (−7.32 mg/dL, 95% CI −9.82, −4.82), triglyceride levels (−18.47 mg/dL, 95% CI −20.13, −16.80) and total cholesterol levels (−6.30 mg/dL, 95% CI −9.59, −3.02), and a greater increase in HDL-cholesterol levels (+3.99 mg/dL, 95% CI 1.22, 6.77). There was no evidence of an effect of the intervention on insulin levels (−2.13 μU/mL, 95% CI −4.86, 0.60). For the studies included in the meta-analysis, heterogeneity was 0% for those included in the sensitivity analysis for BW according to intervention duration (>2 years only, *n* = 2). There was overall large between-study heterogeneity for the other outcomes, with *I*^2^ ranging from 44% (triglycerides) to 98% (insulin and HDL-cholesterol).

### 3.4. Sensitivity Analyses

Due to the small number of studies included in the meta-analysis, we were only able to conduct a sensitivity analysis, by stratifying according to intervention duration (≤2 years and >2 years), for BW. Results indicated a greater beneficial effect of the combination of MD and PA, compared to a control condition, on BW, irrespective of intervention duration (Table 2 and Supplementary Materials I, Figure S1). Greater weight loss occurred in interventions lasting ≤2 years (−6.53 kg, 95% CI −10.86, −2.19) vs. >2 years (−0.59 kg, 95% CI −1.08, −0.10). Due to the large between-study variability with regards to intervention delivery methods and intensity of treatment, as well as the treatments received by the control group, subgroup analyses by stratifying according to these factors was not possible.

### 3.5. Quality Assessment

As all articles included in this review reported on studies that were RCTs, the CONSORT checklist was used to assess their reporting quality. Figure S12 (Supplementary Materials I) illustrates the proportion of included articles that reported each of the CONSORT checklist items. Only nine out of the 37 checklist items were reported by all articles. Fifteen items were reported by ≥50% of the articles, whereas 13 items were reported by <50% of the included articles. All articles reported the scientific abstract and aim(s) of the study, but 33% (*n* = 4) did not include in their title that the study was an RCT [35,36,41,43]. With regards to the methods section of the CONSORT checklist, the majority of articles provided insufficient description for many of the items, apart from participant eligibility criteria, intervention description, statistical methods and interim and additional analyses, which were reported by all articles. Most of the items in the results, discussion and other information of the CONSORT checklist were reported by the majority of the articles, apart from potential harms (reported by 25% of articles), trial registration (reported by 42% of articles) and information about study protocol availability (reported by 17% of articles). The detailed items of the CONSORT checklist that were reported, not reported and not applicable for each article included in this review can be found in Supplementary Materials I, Table S4. Of the 12 articles, nine [34,37,38,39,40,41,42,44,45] reported >50%, two [34,37] >75% and three [35,36,43] reported <50% of the checklist items.

### 3.6. Risk of Bias

**Figure 2:**
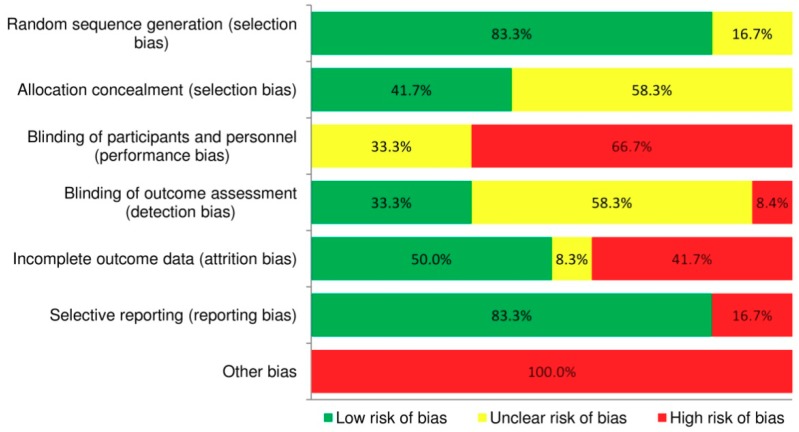
Proportion of included articles assessed as having low, unclear and high risk of bias.

Figure 2 summarises the risk of bias assessment in the included studies. Overall, there was a low risk of bias for random sequence generation and for selective outcome reporting, with 83.3% (*n* = 10) of articles adequately describing selection and reporting bias. Acceptable participant retention and/or reporting of intention-to-treat analyses (attrition bias) were considered to be achieved by 50% of articles. The risk of bias for blinding of participants and personnel, which is challenging for behaviour change interventions, was deemed high for 66.7% (*n* = 8) of articles. More than half of the articles (58.3%, *n* = 7) failed to report whether allocation was concealed or whether outcome assessment was blinded and therefore their risk of bias for these domains was considered unclear. All studies were considered to have high risk of other bias, as they failed to monitor adherence to treatment by using objective measures, which might have led participants to overestimate MD and PA adherence in self-reports. Additionally, the dietary component of the intervention in 10 studies [35,36,37,39,40,41,42,43,45,47] promoted reduced energy, lower carbohydrate intake or low glycaemic index foods alongside the MD, which might have overestimated any effect on metabolic risk outcomes. Details of risk of bias for each included article are illustrated in Supplementary Materials I, Figure S13. Across the 12 articles, risk of bias was considered low, unclear and high for 42%, 25% and 33% of the Cochrane Collaboration tool’s domains, respectively. The funnel plots created in RevMan can be found in Supplementary Materials I, Figures S14–S24. Visual evaluation of these did not suggest evidence of publication bias (note that due to the small number of studies included in the meta-analyses and the high degree of between-study heterogeneity, the triangular region, indicating where 95% of studies should be in the absence of bias, was only created for triglyceride concentrations). Overall, funnel plots did not suggest a possibility for publication bias.

## 4. Discussion

The aim of the current systematic review and meta-analysis of 11 randomised controlled trials was to assess the combined effect of promoting the MD and PA, compared with no treatment, treatment with MD or PA alone, or a different diet and/or PA treatment, on metabolic risk factors in adults. To our knowledge, this is the first paper to systematically evaluate, with a meta-analysis, the effect of concurrently promoting these two integral behavioural components of the traditional Mediterranean lifestyle [6,7] on metabolic risk. This work extends knowledge from earlier meta-analyses that have assessed the effect of the MD on metabolic risk factors defining the metabolic syndrome only [4] and the effect of the MD, when combined with PA, on weight loss [5], by assessing the combined effect of MD and PA as a primary aim and its effects on a wider range of metabolic risk factors that may be important for cardiovascular health. The pooled analysis suggested strong evidence that promoting the MD and PA results in greater beneficial changes in 10 out of 11 risk factors investigated (BW, BMI, WC, SBP, DBP, HOMA-IR index, and blood concentrations of glucose, triglycerides, and total- and HDL-cholesterol), compared to a control condition. In addition, no unfavourable between-group differences in metabolic risk factors were reported in any of the articles, indicating the potential of promoting the MD and PA concurrently for metabolic health.

Several meta-analyses of RCTs have examined the effect of the MD on one or multiple metabolic risk factors, showing overall beneficial effects favouring this dietary pattern. Compared to a control diet, groups receiving a MD intervention in a meta-analysis of 16 RCTs showed greater reductions in BW (−1.75 kg, 95% CI −2.86, −0.64) and BMI (−0.57 kg/m^2^, 95% CI −0.93, −0.21) [5]. A recent meta-analysis of 29 RCTs in 4133 participants reported higher reductions in several metabolic risk factors favouring the intervention, including WC (−0.54 cm, 95% CI −0.77, −0.31), SBP (−0.72 mmHg, 95% CI −1.03, −0.42), DBP (−0.94 mmHg, 95% CI –1.45, −0.44), triglyceride (−0.46 mmol/L, 95% CI −0.72, −0.21) and blood glucose concentrations (−0.50 mmol/L, 95% CI −0.81, −0.20), but no evidence to support an effect on HDL-cholesterol [4]. Similarly, a meta-analysis of 35 RCTs showed a protective effect of the MD on WC (−0.42 cm, 95% CI −0.82, −0.02), HDL-cholesterol (+1.17 mg/dL, 95% CI, 0.38, 1.96), triglycerides (−6.14 mg/dL, 95% CI −10.35, −1.93), SBP (−2.35 mmHg, 95% CI −3.51, −1.18), DBP (−1.58 mmHg, 95% CI −2.02, −1.13), HOMA-IR index (−0.45, 95% CI −0.74, −0.16) and blood glucose (−3.89 mg/dL, 95% CI, −5.84, −1.95) [2]. With regards to PA, a meta-analysis of 29 RCTs showed greater improvements, favouring the intervention group, in triglycerides (−5.31 mg/dL, 95% CI −10.63, −0.89), HDL-cholesterol (+2.32 mg/dL, 95% CI 1.16, 3.87), insulin (−1.03 μU/mL, 95% CI −1.69, −0.37) and HOMA-IR index (−0.3, 95% CI −0.49, −0.11), but no evidence of an effect on total cholesterol, LDL-cholesterol or blood glucose [16]. Another meta-analysis evaluating the effect of resistance training showed a protective effect on SBP (−6.19 mmHg, 95% CI −11.38, −1.00), but weak evidence to support an effect in total-, HDL- and LDL-cholesterol, as well as triglycerides and DBP [13]. Other meta-analyses on different types of PA have shown that combined training (endurance, dynamic and isometric resistance) has no effect on SBP but results in reductions in DBP (−2.2 mmHg, 95% CI, −3.9, −0.48) [14] and that aerobic training results in greater increases in HDL-cholesterol (+2.53 mg/dL, 95% CI 1.36, 3.70) favouring the intervention group [18]. Overall, these meta-analyses suggest a beneficial independent effect of the MD and PA on a variety of metabolic risk factors in adult populations.

Comparisons between the current meta-analysis and findings from earlier pooled analyses of RCTs are hindered by differences in the analyses’ primary aim, as well as population characteristics, study inclusion and exclusion criteria and definitions of the MD and PA in the included studies. Nevertheless, our findings showed that interventions combining the MD and PA lead to comparable or greater between-group improvements in most metabolic risk factors (BW, BMI, WC, SBP, DBP, glucose, HOMA-IR index, triglycerides, and HDL-cholesterol), compared to between-group changes reported in earlier meta-analyses of the MD [2,4,5], as well as meta-analyses of PA (for SBP, DBP, glucose, HOMA-IR index, triglycerides, total-cholesterol and HDL-cholesterol) [13,14,16,18]. Although these comparisons should be interpreted with caution, they suggest a potential beneficial role of concurrently promoting the MD and PA, over and above promoting these lifestyle behaviours separately. This is supported by the findings of a meta-analysis that showed that RCTs promoting the MD led to greater body weight loss when the MD intervention was combined with advice to increase PA (−4.01 kg; 95% CI −5.79, −2.23 kg), compared to MD advice alone (−1.75 kg; 95% CI, −2.86, −0.64 kg) [5].

None of the studies included in the current systematic review compared the combined effect of promoting the MD and PA against a control group receiving a PA intervention only, which would help draw more conclusive evidence on whether promoting these behaviours concurrently leads to greater metabolic effects compared to PA alone. Only one study [43,46] (not included in the meta-analysis due to insufficient data) compared the effect of a (hypocaloric) MD, in combination with moderate-to-high intensity training, against a control group receiving a (hypocaloric) MD only. This study showed that the intervention led to greater beneficial changes in BW, BMI, DBP, triglycerides, insulin and HOMA-IR index, but not WC, SBP, total-, LDL- and HDL-cholesterol or glucose concentrations, compared to the control condition. Despite the plausibility of the hypothesis of a greater beneficial effect when the MD and PA are promoted concurrently, compared to separately, this hypothesis should be tested further by high-quality, well-reported and adequately powered randomised controlled trials. Ideally, these trials should employ three control groups (MD alone, PA alone and no treatment) to establish whether a combined MD and PA intervention exerts greater protective effects than promoting each behaviour alone, and ascertain its true effect on metabolic risk.

Two studies, included in the qualitative synthesis but not the pooled analysis, compared a treatment combining the MD and PA against usual care and found beneficial between-group differences, favouring the intervention group, in half of the metabolic risk factors they assessed, including triglycerides, total- and LDL-cholesterol [34] and BW, BMI, WC, SBP and insulin concentrations [35]. Potential mechanisms through which the traditional MD can exert benefits on metabolic risk include its anti-inflammatory [48] and antioxidant [49] properties, high fibre, n-3 fatty acid, polyphenol and other phytochemical content [50], as well as its role in improving insulin sensitivity [51]. PA has also been linked to improved metabolic health through various biological mechanisms, including regulation of adipokine expression and adipose tissue inflammation, controlling dyslipidaemia via regulation of lipoprotein lipase activity, improving insulin sensitivity [16,52], improving body composition and enhancing endothelial function [52]. It has also been suggested that combining the MD and PA improves cardiovascular risk through beneficial effects on vascular activity [53] and, when coupled with moderate alcohol consumption and not smoking, reductions in general and abdominal adiposity [54]. Although further studies are needed to establish the exact biological mechanisms of any beneficial combined effect [24], evidence to date offers a plausible mechanism of action and indicates the potential of concurrently promoting the MD and PA for metabolic risk reduction.

The studies reviewed varied widely with regards to participant characteristics, sample size, intervention duration, as well as the nature, intensity and delivery methods of the MD and PA intervention and control treatment components, with associated limitations in measures of adherence to treatment. This, in addition to the small number of studies included in the pooled analysis, resulted in high levels of between-study heterogeneity, which, even if similar to earlier meta-analyses of MD [4,5] and PA [13,16] RCTs, leaves uncertainty as to what the true effect of the intervention might be. Despite these differences, results from all studies included in the meta-analysis pointed towards the same direction of effect for all metabolic risk factors. The majority of studies were conducted in Mediterranean countries, which might also reduce the generalisability of the results. There is no reason to believe that any metabolic benefits of promoting the MD and PA would not be transferable outside Mediterranean regions [1]; however, people residing in non-Mediterranean countries have different cultural and dietary habits that might render adhering to the MD component of such an intervention challenging [55,56,57,58], thereby potentially reducing the feasibility of achieving the level of change in MD required to observe the same magnitude of effect. Further studies in non-Mediterranean populations are needed to confirm this.

There was some variation in the quality of reporting among the articles included in the current review, with only nine out of the 37 CONSORT checklist items reported by all articles and only two articles reporting >75% of the checklist items. Many items related to the studies’ methods, such as trial design, sample size, type of randomisation, allocation concealment, implementation and blinding were not sufficiently reported in the majority of the articles. In addition, most studies failed to report any potential harms (or lack of) of their treatment. Based on the aforementioned evidence on biological mechanisms, it is reasonable to hypothesise that the combination of MD and PA would not cause any harm, and some authors might have chosen not to report this checklist item, particularly when considering strict word count guidelines in publishing. However, establishing the safety of any treatment is crucial to inform public health guidance and should be included in reports of intervention studies. Overall, the reporting quality of the included studies hinders the interpretation of the overall findings and highlights the need for future reports of RCTs to conform to current guidelines of reporting [28].

Across the articles included in this systematic review, risk of bias was considered low, unclear and high for 42%, 25% and 33% of domains in the Cochrane Collaboration’s tool [30], respectively. Domains assessed as having unclear risk included allocation concealment and blinding of outcome assessment (58.3% of articles did not report any of these domains). These are important features that authors of future RCTs should include in their study design reports, as RCTs which do not incorporate allocation concealment [59] or conduct outcome assessment in a non-blinded manner [60] have been found to overestimate treatment effects. The lack of blinding of study personnel and participants (66.7% of articles) or its unclear reporting (33.3% of articles) might also have introduced bias, although this is a common challenge in lifestyle interventions or RCTs testing different types of patient management [60,61]. Future RCTs examining the combined effect of the MD and PA on metabolic risk factors could also overcome other sources of bias that the current review assessed as high risk, by using more objective methods to assess adherence to treatment (e.g., accelerometers to assess PA [62] or biomarkers to assess MD adherence [63]), instead of relying on self-reported adherence. Findings of the current risk of bias assessment highlight the need for future RCTs to consider all domains in the Cochrane Collaboration’s tool [30] during the planning phase, and report these clearly in publications to improve the validity of findings of future systematic reviews and meta-analyses.

The strength of the current systematic review is the application of rigorous methodology, conducted in line with the Cochrane handbook [31] and reported following the PRISMA guidelines [25,26], to investigate, for the first time, the combined effect of the MD and PA on a variety of metabolic risk factors. We conducted a comprehensive search of four databases and followed established guidelines for the assessment of both the included studies’ quality reporting [28] and risk of bias [30], as well as systematic review and meta-analysis reporting [25,26]. However, there are several limitations in the present analyses that should be considered when interpreting the findings. We did not exclude studies that combined the MD with other dietary factors as part of the intervention treatment, which likely contributed to the high levels of between-study heterogeneity. As 9 out of the 11 studies promoted the MD along with energy restriction [36,37,39,40,41,42,43,45] and a low carbohydrate [37,39] or low-glycaemic content [35] as part of the dietary component of their intervention, it might be that these additional intervention components, instead of the combination of the MD and PA, were responsible for the observed beneficial effects in metabolic risk factors. Future RCTs planning to establish the combined effect of the MD and PA on metabolic risk should ideally include an additional trial arm where components, such as energy restriction, would be provided in addition to the MD and PA intervention, so that any such intervention effects can be distinguishable. We also cannot exclude the possibility that potentially eligible studies might not have been included in this review due to the pre-set study eligibility criteria (e.g., language restrictions). Finally, the limited number of studies included in this review, in addition to the high between-study heterogeneity observed, did not allow sensitivity analyses to be conducted according to treatment characteristics, intervention duration, sample size, country or control group treatment, for all metabolic risk factors of interest. The sensitivity analysis for BW, stratified by intervention duration, revealed that concurrently promoting the MD and PA led to greater weight loss in interventions lasting ≤2 years, compared to those >2 years, although there was still moderate evidence (*p* = 0.02) to suggest a long-term effect. Nevertheless, more RCTs with longer follow-up periods are needed to confirm the long-term combined effect of the MD and PA on metabolic risk factors.

## 5. Conclusions

Despite the aforementioned limitations, findings from this systematic review with meta-analysis suggest, for the first time, that the MD and PA, when promoted concurrently, lead to beneficial changes in a number of metabolic risk factors. Considering that these lifestyle behaviours formed essential characteristics of the health promoting traditional Mediterranean lifestyle [6,21], and the documented independent effect of the MD [1,2,4,5] and PA [13,14,16,18] on metabolic parameters, their combined promotion may be a useful means to reduce metabolic risk in adult populations. Findings, however, should be interpreted with caution, particularly when considering the small number of studies and the high degree of heterogeneity between the studies included in the current analysis. More high-quality and adequately powered RCTs, reported according to current reporting guidelines [27,30,31] and including objective measures of adherence to treatment, are needed to establish whether promotion of a traditional Mediterranean lifestyle, characterised by adherence to the MD and high levels of PA, should form part of public health recommendations for metabolic risk reduction. In addition, trials with appropriate comparator groups are needed to help confirm the hypothesis that promoting the MD and PA public health guidelines together results in greater health benefits, over and above those acquired separately by the MD and PA. This will also help establish whether future public health interventions promoting the MD and PA independently should include a PA and MD component, respectively, to potentially induce greater metabolic benefits in adult populations.

## Figures and Tables

**Table 1:** Characteristics of included studies.

**Study**	**Country**		**Research Period**		**Setting**		**Population**		***n***			**I/C** **Groups**	**Intervention Period**	**Intervention Group Treatment**			**Control Group Treatment**			
														**Diet**	**PA**		**Diet**		**PA**	
Droste et al. 2013 [34]	LU		2009 to 2011		Neurology department		Outpatients with carotid atherosclerosis		108			53/55	5 months (≤2 years)	Education on MD	Advice on ↑ PA		Usual diet		Usual PA	
Dunn et al. 2014 [35]	AU		NR		University population		Young overweight women		30			15/15	3 months (≤2 years)	Education on ↓ glycaemic index MD and ω-3 capsules	20-min exercise 3 days/week		Usual diet		Usual PA	
Esposito et al. 2003 [40]	IT		1999 to 2002		University Hospital		Obese subjects		120			60/60	2 years (≤2 years)	Education on ↓ energy MD and behavioural counselling	Advice on ↑ PA		Advice on healthy eating		General advice on PA	
Esposito et al. 2004 [39]	IT		2001 to 2004		University Hospital		Outpatients with metabolic syndrome and sedentary lifestyle		180			90/90	2 years (≤2 years)	Education on ↓ energy MD and behavioural counselling	Advice on ↑ PA (≥30 min/day)		Advice on PD		Advice on ↑ PA (≥30 min/day)	
Esposito et al. 2009 [37]	IT		2004 to 2008		Teaching Hospital		Overweight subjects with newly diagnosed T2D (no antihyperglycemic therapy)		215			108/107	4 years (>2 years)	Education on ↓ energy and ↓ CHO MD	Advice on ↑ PA (≥30 min/day)		Education on ↓ energy and LFD		Advice on ↑ PA (≥30 min/day)	
Esposito et al. 2010 [36]	IT		2000 to 2004		Teaching Hospital		Obese subjects with erectile dysfunction and sedentary lifestyle		192			98/94	2 years (≤2 years)	Education on ↓ energy MD	Advice on ↑ PA (≥30 min/day)		Advice on healthy eating		No PA advice	
Esposito et al. 2014 [38] (follow-up of Esposito et al. 2009)	IT		2004 to 2012		Teaching Hospital		Overweight subjects with newly diagnosed T2D (no antihyperglycemic therapy)		201			102/99	6 years (>2 years)	Education on ↓ energy and ↓ CHO MD	Advice on ↑ PA (≥30 min/day)		Education on ↓ energy and LFD		Advice on ↑ PA (≥30 min/day)	
Gomez-Huelgas et al. 2015 [41]	ES		2007 -		Community health centre		Subjects with metabolic syndrome		406			230/176	3 years (>2 years)	Education on ↓ energy MD	Advice on ↑ PA (150 min/week)		Advice on healthy eating		General advice on PA	
Kiechle et al. 2017 [42]	DE		2014 -		Three university hospitals		Subjects with a pathogenic BRCA1 or BRCA2 germline mutation		68			33/35	1 year (≤2 years)	Education on ↓ energy (if needed) MD	Structured, individualised endurance training plan to increase PA to ≥18 MET hours/week		Advice on healthy eating		General advice on PA	
Landaeta-Diaz et al. 2013 [43]	ES		2009 to 2010		University Hospital		Obese subjects with metabolic syndrome		40			20/20	3 months (≤2 years)	Education on ↓ energy MD	Advice and supervised sessions for ↑ PA		Education on ↓ energy MD		No PA advice	
Ortner Hadžiabdić et al. 2016 [44]	HR		2008 to 2012		University Hospital		Obese subjects		84			40/44	1 year (≤2 years)	Education and supervision on MD	Advice on ↑ PA (≥30 min/day)		Education and supervision on LFD		Advice on ↑ PA (≥30 min/day)	
Papandreou et al. 2012 [45]	GR		2008 to 2009		University Hospital		Obese subjects with obstructive sleep apnoea		40			20/20	6 months (≤2 years)	Education on ↓ energy MD	Advice on ↑ PA (≥30 min/day)		Advice on ↓ energy PD		Advice on ↑ PA (≥30 min/day)	
**Study**	**Risk Factors**												**Intervention Group**				**Control Group**			
	**BW**	**WC**	**BMI**	**SBP**	**DBP**	**Insulin**	**HOMA**	**Glu**	**TG**	**LDL**	**HDL**	**TC**	***n***	**Mean Age (SD)**	**Males/Females**		***n***	**Mean Age (SD)**	**Males/Females**	
															***n***	**%**			***n***	**%**
Droste et al. 2013 [34]	• ^b^		• ^b^						• ^b^	• ^b^	• ^b^	• ^b^	53	63.7 (8.1)	37/16	69.8/30.2	55	63.4 (10.6)	35/20	63.6/36.4
Dunn et al. 2014 [35]	• ^a,b^	• ^a,b^	• ^a,b^	• ^a,b^	• ^a,b^	• ^a,b^	• ^a,b^	• ^a,b^	• ^a,b^	• ^a,b^	• ^a,b^	• ^a,b^	15	24.0 (1.0)	0/15	0.0/100.0	15	22.0 (0.6)	0/15	0.0/100.0
Esposito et al. 2003 [40]	•		•	•	•	•	•	•	•		•	•	60	34.2 (4.8)	0/60	0.0/100.0	60	35.0 (5.1)	0/60	0.0/100.0
Esposito et al. 2004 [39]	•	•	•	•	•	•	•	•	•		•	•	90	44.3 (6.4)	47/43	552.2/47.8	90	43.5 (5.9)	50/40	56/44
Esposito et al. 2009 [37]	•	•	•	•	•	•	•	•	•		•	•	108	52.4 (11.2)	54/54	50.0/50.0	107	51.9 (10.7)	52/55	48.6/51.4
Esposito et al. 2010 [36]	•					• ^b^		• ^b^				• ^a,b^	52	43.5 (4.8) ^c^	52/0	100.0/0.0	50	43.0 (5.1) ^d^	50/0	100.0/0.0
Esposito et al. 2014 [38] (follow-up of Esposito et al. 2009)	• ^b^	• ^a,b^		• ^a,b^	• ^a,b^			• ^a,b^	• ^a,b^		• ^a,b^	• ^a,b^	108	52.4 (11.2)	54/54	50.0/50.0	107	51.9 (10.7)	52/55	48.6/51.4
Gomez-Huelgas et al. 2015 [41]	•	•	•	•	•				•	•	•	•	298	53.9 (14.3)	165/133	55.4/44.6	303	53.7 (13.7)	166/137	54.8/45.2
Kiechle et al. 2017 [42]			• ^d^										33	45 (30–51) ^d^	0/33	0.0/100.0	35	34 (26–46)	0/35	0.0/100.0
Landaeta-Diaz et al. 2013 [43]	• ^a,b^	• ^a,b^	• ^a,b^	• ^a,b^	• ^a,b^	• ^a,b^	• ^a,b^	• ^a,b^	• ^a,b^	• ^a,b^	• ^a,b^	• ^a,b^	20	59.1 (1.2)	7/13	35.0/65.0	20	57.2 (0.9)	5/15	25.0/75.0
OrtnerHadžiabdić et al. 2016 [44]	• ^b^												63	46.2 (12.7)	19/44	30.2/69.8	61	49.0 (12.1)	13/48	21.3/78.7
Papandreou et al. 2012 [45]	•	•	•										20	52.2 (10.5)	17/3	85.0/15.0	20	45.8 (14.2)	17/3	85.0/15.0

AU, Australia; BMI, body mass index; BW, body weight; C, control; CHO, carbohydrates; DBP, diastolic blood pressure; DE, Germany; ES, Spain; Glu, glucose; GR, Greece; HDL, high density lipoprotein cholesterol; HOMA, homeostatic model assessment of insulin resistance; HR, Croatia; I, intervention; IT, Italy; LDL, low density lipoprotein cholesterol; LFD, low-fat diet; LU, Luxembourg; MD, Mediterranean Diet; MET, metabolic equivalent; NR, not reported; PA, Physical Activity; PD, prudent diet; SBP, systolic blood pressure; SD, standard deviation; T2D, type 2 diabetes; TC, total cholesterol; TG, triglycerides; WC, waist circumference. Bullets indicate the risk factors reported by each article. ^a^ *p*-value not reported; ^b^ Means, standard deviations or confidence intervals are not reported; ^c^ Median (range) is reported. ^d^ Obtained from [47]. Due to ^a,b,c^, these studies could not be included in the meta-analysis. “↓” means decreased; “↑” means increased.

**Table 2:** Combined effect of the Mediterranean diet and physical activity on metabolic risk factors.

Outcome or Subgroup	Studies	Participants	Effect Estimate (MD, 95% CI)	*p*-Value	*I* ^2^
Body weight (kg) ^a^	6	1153	−3.68 (−5.48, −1.89)	<0.001	95%
Up to 2 years of intervention	4	532	−6.53 (−10.86, −2.19)	0.003	93%
More than 2 years of intervention	2	621	−0.59 (−1.08, −0.10)	0.020	0%
Waist circumference (cm)	4	701	−1.62 (−2.58, −0.66)	<0.001	77%
Body mass index (kg/m^2^)	5	825	−0.64 (−1.10, −0.18)	<0.001	82%
Systolic blood pressure (mm Hg)	4	765	−0.83 (−1.57, −0.09)	<0.001	95%
Diastolic blood pressure (mm Hg)	4	765	−1.96 (−2.57, −1.35)	<0.001	48%
Insulin (μU/mL)	3	379	−2.13 (−4.86, 0.60)	0.130	98%
HOMA-IR index	3	379	−0.90 (−1.22, −0.58)	<0.001	74%
Glucose (mg/dL)	3	379	−7.32 (−9.82, −4.82)	<0.001	74%
Triglycerides (mg/dL)	4	785	−18.47 (−20.13, −16.80)	<0.001	44%
HDL-cholesterol (mg/dL)	4	785	+3.99 (1.22, 6.77)	<0.001	98%
Total cholesterol (mg/dL)	4	785	−6.30 (−9.59, −3.02)	<0.001	63%

CI; confidence intervals; HDL, high density lipoprotein cholesterol; HOMA-IR, homeostatic model assessment of insulin resistance; MD, mean difference. Findings are based on random-effects meta-analysis (inverse variance), apart from triglycerides (fixed effects). *I*^2^ represents the magnitude of heterogeneity. ^a^ Sensitivity analysis, with studies stratified according to intervention duration.

## Supplementary Materials

The following are available online at http://www.mdpi.com/2072-6643/10/11/1577/s1, Supplementary Material I, Table S1: PRISMA checklist; Table S2: Table of excluded articles; Table S3: Summary of the findings (between-group differences) from the articles included in the systematic review for each metabolic risk factor; Table S4: Reporting quality of the included articles, based on the CONSORT 2010 checklist; Figures S1–S11: Forest plots of randomised controlled trials evaluating the combined effect of the Mediterranean diet and physical activity on metabolic risk factors; Figure S12: Proportion of included articles reporting each of the CONSORT checklist items; Figure S13: Detailed risk of bias for each included article; Figures S14–S24: Funnel plots of randomised controlled trials evaluating the combined effect of the Mediterranean diet and physical activity on metabolic risk factors. Supplementary Material II: Search strategy.

## Note

References 36,37,39–41,45 indicate research reports included in the meta-analysis.
