# Supplementary material for: The Combined Effect of Promoting the Mediterranean Diet and Physical Activity on Metabolic Risk Factors in Adults: A Systematic Review and Meta-Analysis of Randomised Controlled Trials
Source: Nutrients. 2018 Oct 25;10(11):1577. doi: 10.3390/nu10111577 (PMC6267322; doi:10.3390/nu10111577)
Supplement: Supplementary file 1 [file nutrients-10-01577-s001.zip › nutrients-372737-supplementary/nutrients-372737-proof done-supplementary I.pdf]

Supplementary Materials I, Table S1. PRISMA checklist

Section/topic	#	Checklist item	Reported on page #
**TITLE**			
Title	1	Identify the report as a systematic review, meta-analysis, or both.	1
**ABSTRACT**			
Structured summary	2	Provide a structured summary including, as applicable: background; objectives; data sources; study eligibility criteria, participants, and interventions; study appraisal and synthesis methods; results; limitations; conclusions and implications of key findings; systematic review registration number.	1
**INTRODUCTION**			
Rationale	3	Describe the rationale for the review in the context of what is already known.	2
Objectives	4	Provide an explicit statement of questions being addressed with reference to participants, interventions, comparisons, outcomes, and study design (PICOS).	2-3
**METHODS**			
Protocol and registration	5	Indicate if a review protocol exists, if and where it can be accessed (e.g., Web address), and, if available, provide registration information including registration number.	3
Eligibility criteria	6	Specify study characteristics (e.g., PICOS, length of follow-up) and report characteristics (e.g., years considered, language, publication status) used as criteria for eligibility, giving rationale.	3
Information sources	7	Describe all information sources (e.g., databases with dates of coverage, contact with study authors to identify additional studies) in the search and date last searched.	4
Search	8	Present full electronic search strategy for at least one database, including any limits used, such that it could be repeated.	4 and Supplementary Materials II
Study selection	9	State the process for selecting studies (i.e., screening, eligibility, included in systematic review, and, if applicable, included in the meta-analysis).	4, 5
Data collection process	10	Describe method of data extraction from reports (e.g., piloted forms, independently, in duplicate) and any processes for obtaining and confirming data from investigators.	4
Data items	11	List and define all variables for which data were sought (e.g., PICOS, funding sources) and any assumptions and simplifications made.	3, 5
Risk of bias in individual studies	12	Describe methods used for assessing risk of bias of individual studies (including specification of whether this was done at the study or outcome level), and how this information is to be used in any data synthesis.	4-5
Summary measures	13	State the principal summary measures (e.g., risk ratio, difference in means).	5
Synthesis of results	14	Describe the methods of handling data and combining results of studies, if done, including measures of consistency (e.g., I^2) for each meta-analysis.	5

Section/topic	#	Checklist item	Reported on page #
Risk of bias across studies	15	Specify any assessment of risk of bias that may affect the cumulative evidence (e.g., publication bias, selective reporting within studies).	4-5
Additional analyses	16	Describe methods of additional analyses (e.g., sensitivity or subgroup analyses, meta-regression), if done, indicating which were pre-specified.	5
**RESULTS**			
Study selection	17	Give numbers of studies screened, assessed for eligibility, and included in the review, with reasons for exclusions at each stage, ideally with a flow diagram.	5 and<br>Figure 1; Table S2
Study characteristics	18	For each study, present characteristics for which data were extracted (e.g., study size, PICOS, follow-up period) and provide the citations.	6-7 and<br>Tables 1; S3
Risk of bias within studies	19	Present data on risk of bias of each study and, if available, any outcome level assessment (see item 12).	12 and<br>Figure S13
Results of individual studies	20	For all outcomes considered (benefits or harms), present, for each study: (a) simple summary data for each intervention group (b) effect estimates and confidence intervals, ideally with a forest plot.	10 and<br>Tables 2; S3<br>Figures S1-S11
Synthesis of results	21	Present results of each meta-analysis done, including confidence intervals and measures of consistency.	10 and<br>Table 2<br>Figures S1-S11
Risk of bias across studies	22	Present results of any assessment of risk of bias across studies (see Item 15).	12 and<br>Figure 2
Additional analysis	23	Give results of additional analyses, if done (e.g., sensitivity or subgroup analyses, meta-regression [see Item 16]).	10 and<br>Table 2<br>Figure S1
**DISCUSSION**			
Summary of evidence	24	Summarize the main findings including the strength of evidence for each main outcome; consider their relevance to key groups (e.g., healthcare providers, users, and policy makers).	13-15, 16
Limitations	25	Discuss limitations at study and outcome level (e.g., risk of bias), and at review-level (e.g., incomplete retrieval of identified research, reporting bias).	15-16
Conclusions	26	Provide a general interpretation of the results in the context of other evidence, and implications for future research.	14-16
**FUNDING**			
Funding	27	Describe sources of funding for the systematic review and other support (e.g., supply of data); role of funders for the systematic review.	17

From: Moher D, Liberati A, Tetzlaff J, Altman DG, The PRISMA Group (2009). Preferred Reporting Items for Systematic Reviews and Meta-Analyses: The PRISMA Statement. PLoS Med 6(7): e1000097. doi:10.1371/journal.pmed1000097

For more information, visit: www.prisma-statement.org.

Supplementary Materials I, Table S2. Table of excluded articles (n=109)

Author	Title	Reason
Agah Heris, *et al.*	Lipid Profile Improvement after Four Group Psychological Interventions in Combination to Nutritional and Physical Activity Instructing Among Overweight and Obese Individuals	Ineligible type of intervention
Aizawa, *et al.*	Effects of lifestyle modification on central artery stiffness in metabolic syndrome subjects with pre-hypertension and/or pre-diabetes	No control group
Alkhatib, *et al.*	Effects of exercise training and Mediterranean diet on vascular risk reduction in post-menopausal women	Ineligible outcomes
Alkhatib, *et al.*	Long-term cardiorespiratory effects of Mediterranean diet and exercise training intervention in sedentary older participants	Ineligible outcomes
Alkhatib, *et al.*	Reduction in post-menopausal related vascular dysfunction through exercise and Mediterranean diet	Conference abstract
Andreoli, *et al.*	Effect of a moderately hypoenergetic Mediterranean diet and exercise program on body cell mass and cardiovascular risk factors in obese women	No control group
Aragon, *et al.*	The influence of exercise and diet on gastrointestinal peptides circulating levels: Appetite and energy homeostasis	Ineligible outcomes
Augustin, *et al.*	Low glycemic index diet, exercise and vitamin D to reduce breast cancer recurrence (DEDiCa): design of a clinical trial	Conference abstract
Barrera, *et al.*	Multiple-behavior-change interventions for women with type 2 diabetes	Ineligible study design
Bautista-Castano, *et al.*	Variables predictive of adherence to diet and physical activity recommendations in the treatment of obesity and overweight, in a group of Spanish subjects	Ineligible study design
Bekkouche, *et al.*	The Mediterranean diet adoption improves metabolic, oxidative, and inflammatory abnormalities in Algerian metabolic syndrome patients.	Conference abstract
Bigford, *et al.*	A population-relevant lifestyle-intensive intervention for diabetes prevention after SCI	Conference abstract
Boidin, *et al.*	Effect of aquatic interval training with Mediterranean diet counseling in obese patients: Results of a preliminary study	Ineligible study design
Bonfanti, *et al.*	Effect of two hypocaloric diets and their combination with physical exercise on basal metabolic rate and body composition	Other language
Cabrera Sierra, *et al.*	Lifestyle intervention clinical trial in a labour population with high cardiovascular risk: 24 months follow-up preliminary results	Conference abstract
Carey, *et al.*	A low-glycemic nutritional fitness program to reverse metabolic syndrome in professional firefighters: results of a pilot study	No control group
Catalano, *et al.*	Mediterranean diet and physical activity: An intervention study. Does olive oil exercise the body through the mind?	No control group
Caudwell, *et al.*	Exercise alone is not enough: weight loss also needs a healthy (Mediterranean) diet?	No control group
Chair, *et al.*	An Indo Mediterranean diet was more effective than a conventional prudent diet in reducing coronary artery disease risk factors and events	Ineligible type of intervention
Connolly, *et al.*	Outcomes of an integrated community-based nurse-led cardiovascular disease prevention programme	No control group
Corbalán, *et al.*	Effectiveness of cognitive-behavioral therapy based on the Mediterranean diet for the treatment of obesity	No control group
Dalle Grave, *et al.*	Personalized multistep cognitive behavioral therapy for obesity	Ineligible study design
Dalzill, *et al.*	Intensive Lifestyle Intervention Improves Cardiometabolic and Exercise Parameters in Metabolically Healthy Obese and Metabolically Unhealthy Obese Individuals	No control group
Davis, *et al.*	A Mediterranean diet lowers blood pressure and improves endothelial function: results from the MedLey randomized intervention trial.	Ineligible outcomes
de Barros, *et al.*	Realistic changes in monounsaturated fatty acids and soluble fibers are able to improve glucose metabolism	No control group
de Gregorio, *et al.*	Genistein Supplementation and Cardiac Function in Postmenopausal Women with Metabolic Syndrome: Results from a Pilot Strain-Echo Study	Ineligible study design
De Lorenzo, *et al.*	Mediterranean meal versus Western meal effects on postprandial ox-LDL, oxidative and inflammatory gene expression in healthy subjects: a randomized controlled trial for nutrigenomic approach in cardiometabolic risk	Ineligible outcomes
Droste, *et al.*	Lifestyle counseling in patients with carotid arteriosclerosis from Luxemburg should focus more on the reduction of sugar, sodium and saturated fat consumption	Another report of an included study

Dukatz, *et al.*	Feasibility assessment on a lifestyle intervention in healthy and diseased BRCA 1/2 mutation carriers	Conference abstract
Embree, *et al.*	Successful long-term weight loss among participants with diabetes receiving an intervention promoting an adapted Mediterranean-style dietary pattern: the Heart Healthy Lenoir Project	No control group
Endevelt, *et al.*	Diabetes prevention program in a Mediterranean environment: Individual or group therapy? An effectiveness evaluation	Ineligible type of intervention
Fernandez, *et al.*	Moderate-to-high-intensity training and a hypocaloric Mediterranean diet enhance endothelial progenitor cells and fitness in subjects with the metabolic syndrome	Ineligible outcomes
Finocchiaro, *et al.*	Effect of specific educational program on dietary change and weight loss in breast-cancer survivors	No control group
García Almeida, *et al.*	Changes in the total energy expenditure with maintenance of free fat mass, after a physical activity and nutritional program, in overweight and obese patients: Metabolic monitoring of physical activity	Conference abstract
García Almeida, *et al.*	Changes in trans fatty acids intake, body composition and physical activity in a cardiac rehabilitation program. One year of follow up	Conference abstract
García-Casares, *et al.*	Brain Functional Connectivity Is Modified by a Hypocaloric Mediterranean Diet and Physical Activity in Obese Women	No control group
Garcia-Toro, *et al.*	Metabolic syndrome improvement in depression six months after prescribing simple hygienic-dietary recommendations	Conference abstract
Gayda, *et al.*	Effects of an intensive lifestyle intervention including high-intensity interval training on cardiometabolic, body composition and exercise parameters in metabolically non-healthy obese and metabolically healthy but obese patients	Conference abstract
Gayda, *et al.*	Eighteen months of Mediterranean diet counseling and high intensity interval training improved and maintained body composition, cardiometabolic and exercise parameters in obese patients	Conference abstract
Gelli, *et al.*	Effect of a counseling-supported treatment with the Mediterranean diet and physical activity on the severity of the non-alcoholic fatty liver disease	No control group
Gepner, *et al.*	Intramyocellular triacylglycerol accumulation across weight loss strategies; Sub-study of the CENTRAL trial	Ineligible type of intervention
Graffy, *et al.*	The effect of weight change on breast adipose and dense tissue	Conference abstract
Heris, *et al.*	Lipid Profile Improvement after Four Group Psychological Interventions in Combination to Nutritional and Physical Activity Instructing Among Overweight and Obese Individuals	Ineligible type of intervention
Ijzelenberg, *et al.*	The effect of a comprehensive lifestyle intervention on cardiovascular risk factors in pharmacologically treated patients with stable cardiovascular disease compared to usual care: a randomised controlled trial	Conference abstract
Jayo-Montoya, *et al.*	Effects of different high intensity aerobic interval training programs with Mediterranean diet recommendations in post-myocardial infarct patients: Preliminary results of INTERFARCT controlled trials	Conference abstract
Jeejeebhoy, *et al.*	Family physician-led, team-based, lifestyle intervention in patients with metabolic syndrome: results of a multicentre feasibility project	No control group
Jilcott, *et al.*	Examining the association between Intervention-Related Changes in Diet, Physical Activity and Weight as Moderated by the Food and Physical Activity Environments among Rural, Southern Adults	Ineligible study design
Kadda, *et al.*	Lifestyle intervention and one-year prognosis of patients following open heart surgery: a randomised clinical trial	Conference abstract
Katsagoni, *et al.*	A Mediterranean diet versus a Mediterranean lifestyle intervention in patients with nonalcoholic fatty liver disease (NAFLD): Preliminary results from an ongoing clinical	Conference abstract
Katsagoni, *et al.*	Improvement of metabolic syndrome after intervention based on Mediterranean diet in patients with non-alcoholic fatty liver disease (NAFLD): A randomised-controlled clinical trial	Conference abstract
Katsarou, *et al.*	Stress management and dietary counseling in hypertensive patients: a pilot study of additional effect	Conference abstract
Keyserling, *et al.*	A community-based lifestyle and weight loss intervention promoting a Mediterranean-style diet pattern evaluated in the stroke belt of North Carolina: the Heart Healthy Lenoir Project	Ineligible study design
Klonizakis, *et al.*	Long-term effects of an exercise and Mediterranean diet intervention in the vascular function of an older, healthy population	Ineligible outcomes

Klonizakis, *et al.*	Mediterranean diet- and exercise-induced improvement in age-dependent vascular activity	Ineligible outcomes
Lamb, *et al.*	Nutritional supplementation of hop rho iso-alpha acids, berberine, vitamin D-3, and vitamin K-1 produces a favorable bone biomarker profile supporting healthy bone metabolism in postmenopausal women with metabolic syndrome	Ineligible type of intervention
Lapierre, *et al.*	Eighteen months of intense lifestyle intervention including high intensity interval training improved and maintained body composition, cardiometabolic risk and exercise parameters in obese patients	Conference abstract
La Torre, *et al.*	Improving knowledge and behaviors on diet and physical activity: results of a pilot randomized field trial	Ineligible population
Lehmann, *et al.*	Effects of lifestyle modification on coronary artery calcium progression and prognostic factors in coronary patients-3-Year results of the randomized SAFE-LIFE trial	Ineligible type of intervention
Lerman, *et al.*	Subjects with elevated LDL cholesterol and metabolic syndrome benefit from supplementation with soy protein, phytosterols, hops rho iso-alpha acids, and Acacia nilotica	Conference abstract
Llaneza, *et al.*	Soy isoflavones improve insulin sensitivity without changing serum leptin among postmenopausal women	Ineligible type of intervention
Llaneza, *et al.*	Soy isoflavones, Mediterranean diet, and physical exercise in postmenopausal women with insulin resistance	Ineligible type of intervention
Malaguti, *et al.*	High-protein-PUFA supplementation, red blood cell membranes, and plasma antioxidant activity in volleyball athletes	Ineligible type of intervention
Marcus, *et al.*	Multidisciplinary Treatment of the Metabolic Syndrome Lowers Blood Pressure Variability Independent of Blood Pressure Control	No control group
Marquis-Gravel, *et al.*	Intensive lifestyle intervention including high-intensity interval training program improves insulin resistance and fasting plasma glucose in obese patients	No control group
Marquis-Gravel, *et al.*	Intensive lifestyle program combining Mediterranean diet counseling and high intensity interval training improves glycemic control, cardiometabolic and fitness parameters in obese patients	Conference abstract
Martínez-Delgado, *et al.*	Cardiovascular health education intervention in the Prison of Soria	Ineligible study design
McManus, *et al.*	A randomized controlled trial of a moderate-fat, low-energy diet compared with a low fat, low-energy diet for weight loss in overweight adults	Ineligible type of intervention
Michalsen, *et al.*	Effects of lifestyle modification on the progression of coronary atherosclerosis, autonomic function, and angina - The role of GNB3 C825T polymorphism	Ineligible type of intervention
Middleton, *et al.*	Implementing a Mediterranean diet intervention into a RCT: Lessons learned from a non-Mediterranean based country	Ineligible study design
Noites, *et al.*	Effects of a Phase IV Home-Based Cardiac Rehabilitation Program on Cardiorespiratory Fitness and Physical Activity	Ineligible type of intervention
Orazio, *et al.*	Evaluation of dietetic advice for modification of cardiovascular disease risk factors in renal transplant recipients	Ineligible type of intervention
Panunzio, *et al.*	Randomized, controlled nutrition education trial promotes a Mediterranean diet and improves anthropometric, dietary, and metabolic parameters in adults	Conference abstract
Pennathur, *et al.*	Structured lifestyle intervention in patients with the metabolic syndrome mitigates oxidative stress but fails to improve measures of cardiovascular autonomic neuropathy	Ineligible outcomes
Pirri, *et al.*	Insulin resistance is a lasting bio-marker in non-diabetic essential hypertension patients after effective one-year lifestyle changes	Conference abstract
Pop-Busui, *et al.*	Cardiovascular autonomic neuropathy, oxidative stress and lifestyle intervention in subjects with metabolic syndrome	Ineligible outcomes
Raffel, *et al.*	Cardiovascular denervation and oxidative stress in subjects with metabolic syndrome	Ineligible outcomes
Recio-Rodriguez, *et al.*	Short-Term Effectiveness of a Mobile Phone App for Increasing Physical Activity and Adherence to the Mediterranean Diet in Primary Care: A Randomized Controlled Trial (EVIDENT II Study)	Ineligible type of intervention
Renault, *et al.*	The Treatment of Obese Pregnant Women (TOP) study: a randomized controlled trial of the effect of physical activity intervention assessed by pedometer with or without dietary intervention in obese pregnant women	Ineligible outcomes
Renault, *et al.*	Intake of Sweets, Snacks and Soft Drinks Predicts Weight Gain in Obese Pregnant Women: Detailed Analysis of the Results of a Randomised Controlled Trial	Ineligible outcomes

Roca-Rodríguez, *et al.*	Impact of an outpatient cardiac rehabilitation program on clinical and analytical variables in cardiovascular disease	Conference abstract
Rodriguez, *et al.*	Performance of entero-insular axis in an athletic population: diet and exercise influence	Other language
Rubenfire, *et al.*	The metabolic fitness program: Lifestyle modification for the metabolic syndrome using the resources of cardiac rehabilitation	No control group
Shapiro, *et al.*	The psychological impact of a physician delivered intervention for the prevention of cardiovascular disease: The staged nutrition and activity counseling (SNAC) trial.	Conference abstract
Sosner, *et al.*	Net Blood Pressure Reduction Following 9 Months of Lifestyle and High-Intensity Interval Training Intervention in Individuals With Abdominal Obesity	No control group
Štefan, *et al.*	The Relationship between Lifestyle Factors and Body Composition Young Adults	Ineligible study design
Tapsell, *et al.*	Effect of interdisciplinary care on weight loss: a randomised controlled trial	Ineligible type of intervention
Toobert, *et al.*	Long-term effects of the Mediterranean lifestyle program: A randomized clinical trial for postmenopausal women with type 2 diabetes	Ineligible type of intervention
Toobert, *et al.*	Effects of the Mediterranean lifestyle program on multiple risk behaviors and psychosocial outcomes among women at risk for heart disease	Ineligible type of intervention
Toobert, *et al.*	Biologic and quality-of-life outcomes from the Mediterranean Lifestyle Program: A randomized clinical trial	Ineligible type of intervention
Toobert, *et al.*	Enhancing support for health behavior change among women at risk for heart disease: The Mediterranean Lifestyle Trial	Ineligible type of intervention
Toobert, *et al.*	Seven-year follow-up of a multiple-health-behavior diabetes intervention	Ineligible type of intervention
Toobert, *et al.*	Long-term outcomes from a multiple-risk-factor diabetes trial for Latinas: Viva Bien!	Ineligible type of intervention
Toro, *et al.*	Diet and exercise influence on the proteomic profile of an athlete population	Other language
Trovato, *et al.*	Stress, abdominal obesity and intrarenal resistive index in essential hypertension	Ineligible type of intervention
Trovato, *et al.*	Adenovirus-36 seropositivity enhances effects of nutritional intervention on obesity, bright liver, and insulin resistance	Ineligible type of intervention
Trovato, *et al.*	Mediterranean diet and non-alcoholic fatty liver disease: The need of extended and comprehensive interventions	Ineligible study design
Trovato, *et al.*	Lifestyle interventions, insulin resistance, and renal artery stiffness in essential hypertension	Ineligible type of intervention
Tussing-Humphreys, *et al.*	Building research in diet and cognition: The BRIDGE randomized controlled trial.	Conference abstract
Vacca, *et al.*	Revised Mediterranean diet and changes in lifestyle habits are the first step in the treatment of patients with metabolic syndrome	Conference abstract
Valdes, *et al.*	Prevention of type 2 diabetes with Mediterranean diet. Preliminary results from the first year of intervention of the Egabro-Pizarra study	Conference abstract
Vamvakis, *et al.*	Beneficial effects of non-pharmacological interventions in the management of essential hypertension	Ineligible study design
van Velden, *et al.*	The short-term influence of a Mediterranean-type diet and mild exercise with and without red wine on patients with the metabolic syndrome	Ineligible study design
Villarini, *et al.*	Effects of the "PreveDi" lifestyle modification trial on metabolic syndrome	Other language
Villarini, *et al.*	Lifestyle and breast cancer recurrences: The DIANA-5 trial	Ineligible type of intervention
Walden, *et al.*	Assessing the incremental benefit of an extended duration lifestyle intervention for the components of the metabolic syndrome	No control group
Walizer, *et al.*	Adherence to a lifestyle intervention program not improved by visual knowledge of carotid intima atherosclerosis	Conference abstract
Westergren , *et al.*	Randomized clinical trial studying effects of a personalized supervised lifestyle intervention program on cardiovascular status in physically inactive healthy volunteers	Ineligible study design
Yahia, *et al.*	Insulinoresistance, inflammation, lecithin: Cholesterol acyltransferase and HDL composition are improved by Mediterranean diet in metabolic syndrome patients	Conference abstract
Yahia, *et al.*	Benefits of a Mediterranean-style diet on insulin resistance, plasma lipids, inflammation and oxidative stress in algerian metabolic syndrome patients	Conference abstract

Supplementary Materials I, Table S3. Summary of the findings (between-group differences) from the articles included in the systematic review for each metabolic risk factor

Study	BW	BMI	WC	SBP	DBP	TG[a]
Droste et al 2013 [34]	-0.8%, P>0.050	-0.8%, P>0.050	-	-	-	-13.2%, P=0.036
Dunn et al 2014 [35]	-3.5 kg, P<0.001	-1.4 kg/m^2, P<0.001	-3.7 cm, P<0.001	-11 mmHg, P<0.050	-4.2 mmHg, P>0.050	-0.35 mmol/L, P<0.050
Esposito et al 2003 [40]	-11 kg (95% CI -14, -8), P<0.001	-4.2 kg/m^2 (95% CI -6.4, -2), P<0.001	-	-2 mmHg (95% CI -3.5, -0.5), P=0.009	-1.7 mmHg (95% CI -3, -0.4), P<0.001	-12 mg/dL (95% CI -18, -5), P=0.040
Esposito et al 2004 [39]	-2.8 kg (95% CI -5.1, -0.5), P<0.001	-0.8 kg/m^2 (95% CI -1.4, -0.2), P=0.010	-2 cm (95% CI -3.5, -0.5), P=0.010	-3 mmHg (95% CI -5, -1), P=0.010	-2 mmHg (95% CI -3.5, -0.5), P=0.030	-19 mg/dL (95% CI -32, -6), P=0.001
Esposito et al 2009 [37]	-0.6 kg (95% CI -1.6, 1.2)	-0.3 kg/m^2 (95% CI -0.9, 0.4)	-0.4 cm (95% CI -0.9, 0.5)	-1.5 mmHg (95% CI -4.5, 1.2)	-1.4 mmHg (95% CI -4.0, 1.8)	-0.21 mmol/L (95% CI -0.36, -0.02)
Esposito et al 2010 [36]	−11.9 kg (95% CI -19, -4.7), P<.001	-	-	-	-	-
Esposito et al 2014 [38]	+0.4 kg (95% CI -0.1, 0.7)	-	-0.7 cm (95% CI -0.16, 0.3)	-1.8 mmHg (95% CI -4.5, 1.0)	-1.5 mmHg (95% CI -4, 1.9)	-12 mg/dL (95% CI -30, 6)
Gomez-Huelgas et al 2015 [41]	-0.5 kg, P>0.050	-0.19 kg/m^2, P>0.050	-2.4 cm, P<0.001	-4.9 mmHg, P=0.004	-4.4 mmHg, P<0.001	-10 mg/dL, P>0.050
Kiechle et al 2017 [42]	-	+1.6 kg/m^2, P=0.115	-	-	-	-
Landaeta-Diaz et al 2013 [43], Fernandez et al 2012 [44]	-2.98 kg, P<0.050	-1.2 kg/m^2, P<0.050	-0.5 cm, P>0.050	-3.3 mmHg, P>0.050	-8.1 mmHg, P<0.050	-44.03 mg/dL, P<0.050
Ortner Hadziabdic et al 2016 [44]	-3.63 kg, P=0.829	-	-	-	-	-
Papandreou et al 2012 [45]	-1.7 kg, P=0.162	-0.7 kg/m^2, P=0.102	-3 cm, P=0.013	-	-	-

Table S3 (continued)

Study	HDL[b]	LDL[b]	TC[b]	Insulin[c]	Glucose[d]	HOMA-IR
Droste et al 2013 [34]	-0.1%, P>0.050	-7%, P=0.029	-6%, P=0.023	-	-	-
Dunn et al 2014 [35]	-0.11 mmol/L, P>0.050	-0.03 mmol/L, P>0.050	-0.32 mmol/L, P>0.050	-6.8 µIU/mlL, P<0.050	-0.01 mmol/L, P>0.050	-1.34, P>0.050
Esposito et al 2003 [40]	+4 mg/dL (95% CI 2, 6), P=0.020	-	-4 mg/dL (95% CI -12, -4), P=0.130	-3 µU/mL (95% CI -5, -1), P=0.009	-7 mg/dL (95% CI -9, -5), P<0.001	-0.9 (95% CI -1.3, -0.5), P=0.008
Esposito et al 2004 [39]	+3 mg/dL (95% CI 0.8, 5.2), P=0.030	-	-9 mg/dL (95% CI -17, -1), P=0.020	-3.5 µU/mL (95% CI -6.1, -1.7), P=0.010	-6 mg/dL (95% CI -11, -2), P<0.001	-1.1 (95% CI -1.9, -0.3), P<0.001
Esposito et al 2009 [37]	+0.07 mmol/L (95% CI 0.02, 0.14)	-	-0.15 mmol/L (95% CI -0.39, 0.05)	-4.2 pmol/L (95% CI -10.7, 3.4)	-0.9 mmol/L (95% CI -1.6, -0.2)	-0.6 (95% CI -1.1, -0.1)
Esposito et al 2010 [36]	-	-	-4 mg/dL, P>0.050	-1.8 µU/mL, P=0.040	-1.9 mg/dl, P=0.040	-
Esposito et al 2014 [38]	+4.7 mg/dL (95% CI 0.2, 9.1)	-	-4 mg/dL (95% CI -10, 2)	-	-10 mg/dL (-25, 5)	-
Gomez-Huelgas et al 2015 [41]	+2 mg/dL, P=0.050	-2 mg/dL, P>0.050	-1 mg/dL, P>0.050	-	-	-
Kiechle et al 2017 [42]	-	-	-	-	-	-
Landaeta-Diaz et al 2013 [43], Fernandez et al 2012 [44]	0.0 mg/dL, P>0.050	-1.4 mg/dL, P>0.050	-4.9 mg/dL, P>0.050	-27.9pmol/L, P<0.050	0.0 mmol/L, P>0.050	-0.95, P<0.050
Ortner Hadziabdic et al 2016 [44]	-	-	-	-	-	-
Papandreou et al 2012 [45]	-	-	-	-	-	-

BMI, body mass index; BW, body weight; DBP, diastolic blood pressure; HDL, high density lipoprotein cholesterol; HOMA-IR, homeostatic model assessment of insulin resistance; LDL, low density lipoprotein cholesterol; SBP, systolic blood pressure; TC, total cholesterol; TG, triglycerides; WC, waist circumference.

[a] Triglyceride concentrations were transformed from mmol/L to mg/dL by multiplying with 88.57; [b] Total, HDL- and LDL- cholesterol concentrations were transformed from mmol/L to mg/dL by multiplying with 38.67; [c] Insulin concentrations were transformed from pmol/L to µU/mL by multiplying with 0.144; [d] Glucose concentrations were transformed from mmol/L to mg/dL by multiplying with 18.

https://www.ncbi.nlm.nih.gov/books/NBK83505/; http://www.endmemo.com/medical/unitconvert/Insulin.php; https://www.diabetes.co.uk/blood-sugar-converter.html.

Supplementary Materials I, Table S4. Reporting quality of the included articles, based on the CONSORT 2010 checklist

Study	Items reported, total reported (%)	Items not reported, total not reported (%)	Not applicable items, total (%)
Droste et al 2013 [34]	1a, 1b, 2a, 2b, 3a, 4a, 4b, 5, 6a, 7a, 8a, 8b, 9, 10, 11b, 12a, 12b, 13a, 13b, 14a, 15, 16, 17a, 18, 20, 22, 23, 25 **(76%)**	3b, 6b, 19, 21, 24 **(14%)**	7b, 11a, 14b, 17b **(10%)**
Dunn et al 2014 [35]	1b, 2a, 2b, 4a, 5, 8a, 11b, 12a, 12b, 13b, 17a, 18, 20, 22, 25 **(41%)**	1a, 3a, 3b, 4b, 6a, 6b, 7a, 8b, 9, 10, 11a, 13a, 14a, 15, 16, 19, 21, 23, 24 **(51%)**	7b, 14b, 17b **(8%)**
Esposito et al 2003 [40]	1a, 1b, 2a, 2b, 3a, 4a, 4b, 5, 8a, 9, 11a, 11b, 12a, 12b, 13a, 14a, 15, 16, 17a, 18, 22 **(57%)**	3b, 6a, 6b, 7a, 8b, 10, 13b, 19, 20, 21, 23, 24, 25 **(35%)**	7b, 14b, 17b **(8%)**
Esposito et al 2004 [39]	1a, 1b, 2a, 2b, 4a, 4b, 5, 8a, 9, 11a, 11b, 12a, 12b, 13a, 14a, 15, 16, 17a, 18, 20, 22, 25 **(60%)**	3a, 3b, 6a, 6b, 7a, 8b, 10, 13b, 19, 21, 23, 24 **(32%)**	7b, 14b, 17b **(8%)**
Esposito et al 2009 [37]	1a, 1b, 2a, 2b, 4a, 4b, 5, 6a, 7a, 8a, 8b, 9, 11b, 12a, 12b, 13a, 13b, 14a, 15, 16, 18, 19, 20, 21, 22, 23, 24,25 **(76%)**	3a, 3b, 6b, 10, 11a, 17a, 17b **(19%)**	7b, 14b **(5%)**
Esposito et al 2010 [36]	1b, 2a, 2b, 4a, 4b, 5, 6a, 7a, 8a, 11b, 12a, 12b, 14a, 22 **(35%)**	1a, 3a, 3b, 6b, 8b, 9, 10, 11a, 11b, 13a, 13b, 15, 16, 17a, 18, 19, 20, 21, 23, 24, 25 **(57%)**	7b, 14b, 17b **(8%)**
Esposito et al 2014 [38]	1a, 1b, 2a, 2b, 3b, 4a, 5, 6a, 7b, 8a, 11b, 12a, 12b, 14a, 15, 16, 17b, 18, 20, 21, 22, 23, 25 **(62%)**	3a, 4b, 6b, 7a, 8b, 9, 10, 11a, 13a, 13b, 17a, 19, 24 **(35%)**	14b **(3%)**
Gomez-Huelgas et al 2015 [41]	1b, 2a, 2b, 3a, 4a, 4b, 5, 6a, 11b, 12a, 12b, 13a, 13b, 15, 16, 17a, 18, 20, 21, 22, 25 **(57%)**	1a, 3b, 6b, 7a, 8a, 8b, 9, 10, 11a, 14a, 19, 23, 24 **(35%)**	7b, 14b, 17b **(8%)**
Kiechle et al 2017 [42]	1a, 1b, 2a, 2b, 3a, 4a, 4b, 5, 6a, 7a, 8a, 8b, 12a, 12b, 13a, 13b, 15, 16, 17a, 18, 19, 21, 22, 23, 24 25 **(70%)**	3b, 6b, 9, 10, 14a, 20 **(16%)**	7b, 11a, 11b, 14b, 17b **(14%)**
Landaeta-Diaz et al 2013 [43]	1b, 2a, 2b, 4a, 5, 11b, 12a, 12b, 13a, 13b, 15, 16, 17a, 18, 22, 25 **(43%)**	1a, 3a, 3b, 4b, 6a, 6b, 7a, 8a, 8b, 9, 10, 11a, 14a, 19, 20, 21, 23, 24 **(49%)**	7b, 14b, 17b **(8%)**
Ortner Hadziabdic et al 2016 [44]	1a, 1b, 2a, 2b, 3a, 4a, 5, 6a, 8a, 11b, 12a, 12b, 13a, 13b, 14a, 15, 16, 17a, 18, 20, 21, 22, 25 **(62%)**	3b, 4b, 6b, 7a, 8b, 9, 10, 11a, 19, 23, 24 **(30%)**	7b, 14b, 17b **(8%)**
Papandreou et al 2012 [45]	1a, 1b, 2a, 2b, 4a, 4b, 5, 6a, 8a, 8b, 11b, 12a, 12b, 13a, 13b, 14a, 15, 16, 17a, 18, 19, 20, 21, 22, 23 **(68%)**	3a, 3b, 6b, 7a, 9, 10, 24, 25 **(22%)**	7b, 11a, 14b, 17b **(10%)**

Supplementary Materials I. Forest plots of randomised controlled trials evaluating the combined effect of the Mediterranean diet and physical activity on metabolic risk factors

Study or Subgroup	Intervention Mean	Intervention SD	Intervention Total	Control Mean	Control SD	Control Total	Weight	Mean Difference IV, Random, 95% CI
**1.1.1 Up to 24 months of intervention**								
Esposito et al. 2003	-14	8.3837	60	-3	8.3837	60	13.6%	-11.00 [-14.00, -8.00]
Esposito et al. 2004	-4	1.1	90	-1.2	0.6	90	21.8%	-2.80 [-3.06, -2.54]
Esposito et al. 2010	-14	21.6	98	-2	7.4	94	9.1%	-12.00 [-16.53, -7.47]
Papandreou et al. 2012	-8.9	3.9	20	-7.2	4.2	20	15.3%	-1.70 [-4.21, 0.81]
**Subtotal (95% CI)**			**268**			**264**	**59.8%**	**-6.53 [-10.86, -2.19]**
Heterogeneity: Tau² = 17.40; Chi² = 44.92, df = 3 (P < 0.00001); I² = 93%								
Test for overall effect: Z = 2.95 (P = 0.003)								
**1.1.2 More than 24 months of intervention**								
Esposito et al. 2009	-3.8	2	108	-3.2	1.9	107	21.5%	-0.60 [-1.12, -0.08]
Gomez-Huelgas et al. 2015	0.4	9.5	230	0.9	6.6	176	18.7%	-0.50 [-2.07, 1.07]
**Subtotal (95% CI)**			**338**			**283**	**40.2%**	**-0.59 [-1.08, -0.10]**
Heterogeneity: Tau² = 0.00; Chi² = 0.01, df = 1 (P = 0.91); I² = 0%								
Test for overall effect: Z = 2.34 (P = 0.02)								
**Total (95% CI)**			**606**			**547**	**100.0%**	**-3.68 [-5.48, -1.89]**
Heterogeneity: Tau² = 3.84; Chi² = 109.70, df = 5 (P < 0.00001); I² = 95%								
Test for overall effect: Z = 4.02 (P < 0.0001)								
Test for subgroup differences: Chi² = 7.12, df = 1 (P = 0.008), I² = 85.9%								

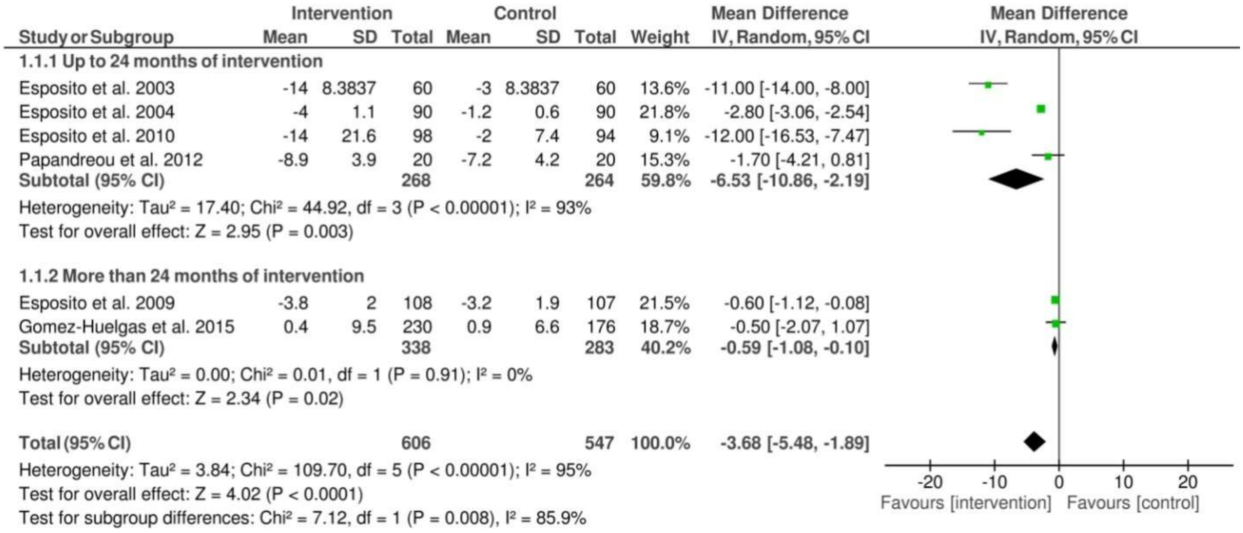

Figure S1. Forest plot for overall body weight estimate and subtotal estimates stratified by intervention duration (up to 24 months and more than 24 months)

Mean differences were pooled using random effects meta-analysis. Squares indicate effect size for each study (mean difference between intervention and control group), with extended lines representing 95% confidence intervals (95% CIs). Diamonds indicate the overall weighted mean effect size. I² indicates between-trial heterogeneity.

One study (Esposito et al, 2010) compared the intervention with a control group receiving advice to change their dietary habits only (education on low-energy MD and advice for ≥30 min/day of PA vs. general advice on healthy eating) [36].

Remaining studies compared the intervention with a control condition receiving advice on both diet and PA. In two studies, the intervention (education on low-energy MD and advice to increase PA, Esposito et al, 2003 and Gomez-Huelgas et al, 2015) was compared with general advice on healthy eating and PA [40, 41]. In two studies, the intervention was compared with a prudent diet and PA (Esposito et al, 2004: advice on a low-energy MD and ≥30 min/day of PA vs. advice on a prudent diet and ≥30 min/day of PA [39]; Papandreou et al, 2012: advice on a low-energy MD and ≥30 min/day of PA vs. advice on a low-energy prudent diet and ≥30 min/day of PA [45]. One study (Esposito et al, 2009) compared the intervention (low-energy, low-carbohydrate MD and ≥30 min/day of PA) to a control group receiving advice on a low-energy, low-fat diet and ≥30 min/day of PA [37].

Study or Subgroup	Intervention Mean	Intervention SD	Intervention Total	Control Mean	Control SD	Control Total	Weight	Mean Difference IV, Random, 95% CI
Esposito et al. 2004	-2	0.5	90	0	0.01	90	37.1%	-2.00 [-2.10, -1.90]
Esposito et al. 2009	-3	1.7	47	-2.6	2	28	28.3%	-0.40 [-1.29, 0.49]
Gomez-Huelgas et al. 2015	0.3	6	230	2.1	6.7	176	22.7%	-1.80 [-3.06, -0.54]
Papandreou et al. 2012	-8.7	3.6	20	-5.7	3.8	20	11.9%	-3.00 [-5.29, -0.71]
**Total (95% CI)**			**387**			**314**	**100.0%**	**-1.62 [-2.58, -0.66]**
Heterogeneity: Tau² = 0.64; Chi² = 13.20, df = 3 (P = 0.004); I² = 77%								
Test for overall effect: Z = 3.31 (P = 0.0009)								

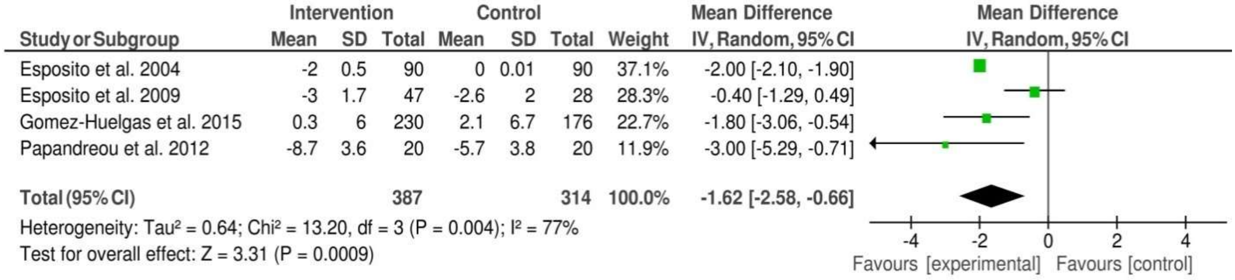

Figure S2. Forest plot for overall waist circumference estimate

Mean differences were pooled using random effects meta-analysis. Squares indicate effect size for each study (mean difference between intervention and control group), with extended lines representing 95% confidence intervals (95% CIs). Diamonds indicate the overall weighted mean effect size. I² indicates between-trial heterogeneity.

All studies compared the intervention with a control condition receiving advice on both diet and PA. One study (Gomez-Huelgas et al, 2015) compared the intervention (education on low-energy MD and advice to increase PA) to general advice on healthy eating and PA [41]. In two studies, the intervention was compared with a prudent diet and PA (Esposito et al, 2004: advice on a low-energy MD and ≥30 min/day of PA vs. advice on a prudent diet and ≥30 min/day of PA [39]; Papandreou et al, 2012: advice on a low-energy MD and ≥30 min/day of PA vs. advice on a low-energy prudent diet and ≥30 min/day of PA [45]. One study (Esposito et al, 2009) compared the intervention (low-energy, low-carbohydrate MD and ≥30 min/day of PA) to a control group receiving advice on a low-energy, low-fat diet and ≥30 min/day of PA [37].

Study or Subgroup	Intervention Mean	Intervention SD	Intervention Total	Control Mean	Control SD	Control Total	Weight	Mean Difference IV, Random, 95% CI
Esposito et al. 2003	-5.2	6.2	60	-1	6.2	60	3.8%	-4.20 [-6.42, -1.98]
Esposito et al. 2004	-1.2	0.3	90	-0.4	0.4	90	31.8%	-0.80 [-0.90, -0.70]
Esposito et al. 2009	-1.2	0.7	50	-0.9	0.6	29	28.6%	-0.30 [-0.59, -0.01]
Gomez-Huelgas et al. 2015	0.12	3.6	230	0.31	2.4	176	21.3%	-0.19 [-0.77, 0.39]
Papandreou et al. 2012	-3.2	1.5	20	-2.5	1.4	20	14.5%	-0.70 [-1.60, 0.20]
**Total (95% CI)**			**450**			**375**	**100.0%**	**-0.64 [-1.10, -0.18]**

Heterogeneity: Tau² = 0.17; Chi² = 22.75, df = 4 (P = 0.0001); I² = 82%
Test for overall effect: Z = 2.73 (P = 0.006)

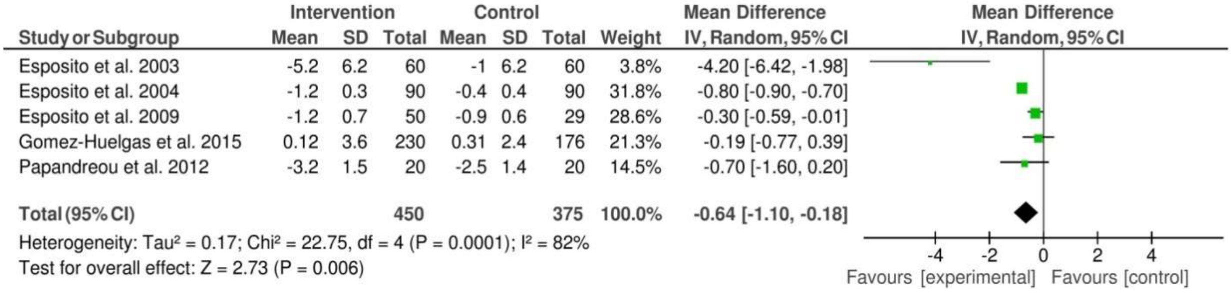

Figure S3. Forest plot for overall body mass index estimate

Mean differences were pooled using random effects meta-analysis. Squares indicate effect size for each study (mean difference between intervention and control group), with extended lines representing 95% confidence intervals (95% CIs). Diamonds indicate the overall weighted mean effect size. I² indicates between-trial heterogeneity.

All studies compared the intervention with a control condition receiving advice on both diet and PA. In two studies, the intervention (education on low-energy MD and advice to increase PA, Esposito et al, 2003 and Gomez-Huelgas et al, 2015) was compared with general advice on healthy eating and PA [40, 41]. In two studies, the intervention was compared with a prudent diet and PA (Esposito et al, 2004: advice on a low-energy MD and ≥30 min/day of PA vs. advice on a prudent diet and ≥30 min/day of PA [39]; Papandreou et al, 2012: advice on a low-energy MD and ≥30 min/day of PA vs. advice on a low-energy prudent diet and ≥30 min/day of PA [45]. One study (Esposito et al, 2009) compared the intervention (low-energy, low-carbohydrate MD and ≥30 min/day of PA) to a control group receiving advice on a low-energy, low-fat diet and ≥30 min/day of PA [37].

Study or Subgroup	Intervention Mean	Intervention SD	Intervention Total	Control Mean	Control SD	Control Total	Weight	Std. Mean Difference IV, Random, 95% CI
Esposito et al. 2003	-3	4.2	60	-1	4.2	60	24.9%	-0.47 [-0.84, -0.11]
Esposito et al. 2004	-4	2	90	-1	1	90	25.0%	-1.89 [-2.24, -1.54]
Esposito et al. 2009	-2.5	2.6	50	-1	1	29	24.0%	-0.69 [-1.16, -0.22]
Gomez-Huelgas et al. 2015	-5.5	15	230	-0.6	19	176	26.1%	-0.29 [-0.49, -0.09]
**Total (95% CI)**			**430**			**355**	**100.0%**	**-0.83 [-1.57, -0.09]**

Heterogeneity: Tau² = 0.53; Chi² = 61.16, df = 3 (P < 0.00001); I² = 95%
Test for overall effect: Z = 2.21 (P = 0.03)

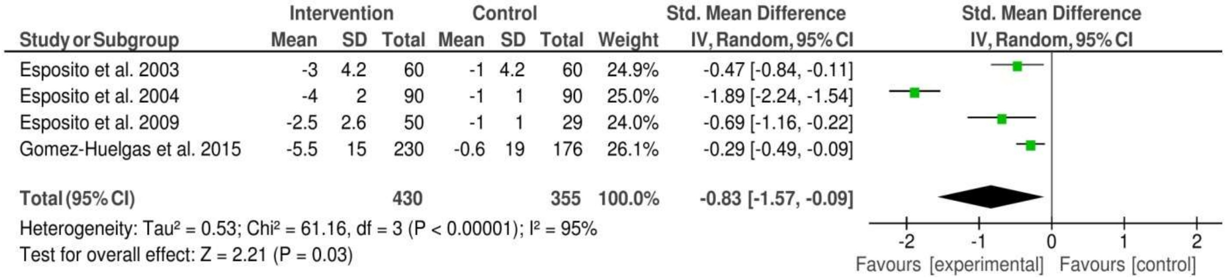

Figure S4. Forest plot for overall systolic blood pressure estimate

Mean differences were pooled using random effects meta-analysis. Squares indicate effect size for each study (mean difference between intervention and control group), with extended lines representing 95% confidence intervals (95% CIs). Diamonds indicate the overall weighted mean effect size. I² indicates between-trial heterogeneity.

All studies compared the intervention with a control condition receiving advice on both diet and PA. In two studies, the intervention (education on low-energy MD and advice to increase PA, Esposito et al, 2003 and Gomez-Huelgas et al, 2015) was compared with general advice on healthy eating and PA [40, 41]. In one study, the intervention was compared with a prudent diet and PA (Esposito et al, 2004: advice on a low-energy MD and ≥30 min/day of PA vs. advice on a prudent diet and ≥30 min/day of PA [39]. One study (Esposito et al, 2009) compared the intervention (low-energy, low-carbohydrate MD and ≥30 min/day of PA) to a control group receiving advice on a low-energy, low-fat diet and ≥30 min/day of PA [37].

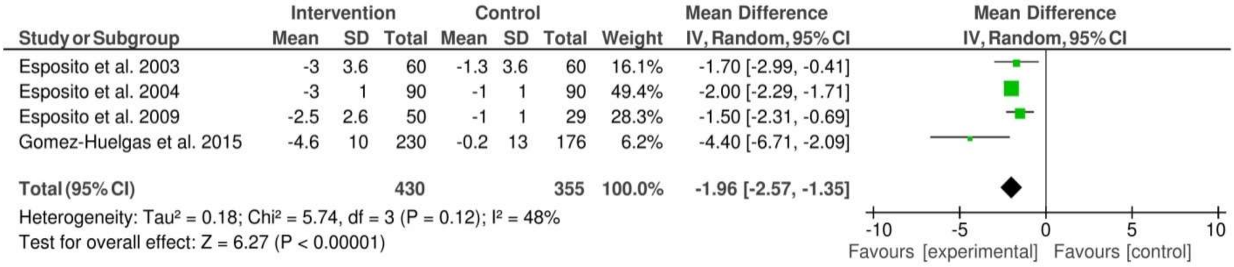

Figure S5. Forest plot for overall diastolic blood pressure estimate

Mean differences were pooled using random effects meta-analysis. Squares indicate effect size for each study (mean difference between intervention and control group), with extended lines representing 95% confidence intervals (95% CIs). Diamonds indicate the overall weighted mean effect size. I^2 indicates between-trial heterogeneity.

All studies compared the intervention with a control condition receiving advice on both diet and PA. In two studies, the intervention (education on low-energy MD and advice to increase PA, Esposito et al, 2003 and Gomez-Huelgas et al, 2015) was compared with general advice on healthy eating and PA [40, 41]. In one study, the intervention was compared with a prudent diet and PA (Esposito et al, 2004: advice on a low-energy MD and ≥30 min/day of PA vs. advice on a prudent diet and ≥30 min/day of PA [39]. One study (Esposito et al, 2009) compared the intervention (low-energy, low-carbohydrate MD and ≥30 min/day of PA) to a control group receiving advice on a low-energy, low-fat diet and ≥30 min/day of PA [37].

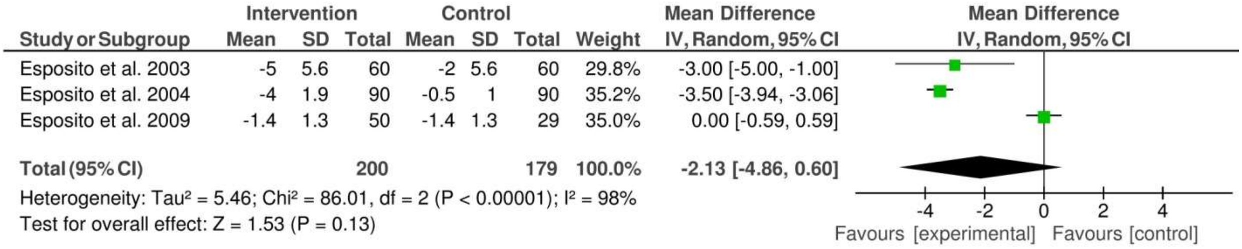

Figure S6. Forest plot for overall insulin concentrations estimate

Mean differences were pooled using random effects meta-analysis. Squares indicate effect size for each study (mean difference between intervention and control group), with extended lines representing 95% confidence intervals (95% CIs). Diamonds indicate the overall weighted mean effect size. I^2 indicates between-trial heterogeneity.

All studies compared the intervention with a control condition receiving advice on both diet and PA. In one study (Esposito et al, 2003), the intervention (education on low-energy MD and advice to increase PA) was compared with general advice on healthy eating and PA [40]. In one study, the intervention was compared with a prudent diet and PA (Esposito et al, 2004: advice on a low-energy MD and ≥30 min/day of PA vs. advice on a prudent diet and ≥30 min/day of PA [39]. One study (Esposito et al, 2009) compared the intervention (low-energy, low-carbohydrate MD and ≥30 min/day of PA) to a control group receiving advice on a low-energy, low-fat diet and ≥30 min/day of PA [37].

Study or Subgroup	Intervention Mean	Intervention SD	Intervention Total	Control Mean	Control SD	Control Total	Weight	Mean Difference IV, Random, 95% CI
Esposito et al. 2003	-1.3	1.1	60	-0.4	1.1	60	27.0%	-0.90 [-1.29, -0.51]
Esposito et al. 2004	-1.2	0.5	90	-0.1	0.2	90	43.7%	-1.10 [-1.21, -0.99]
Esposito et al. 2009	-1.5	1	50	-0.9	0.6	29	29.4%	-0.60 [-0.95, -0.25]
**Total (95% CI)**			**200**			**179**	**100.0%**	**-0.90 [-1.22, -0.58]**

Heterogeneity: Tau² = 0.06; Chi² = 7.57, df = 2 (P = 0.02); I² = 74%
Test for overall effect: Z = 5.57 (P < 0.00001)

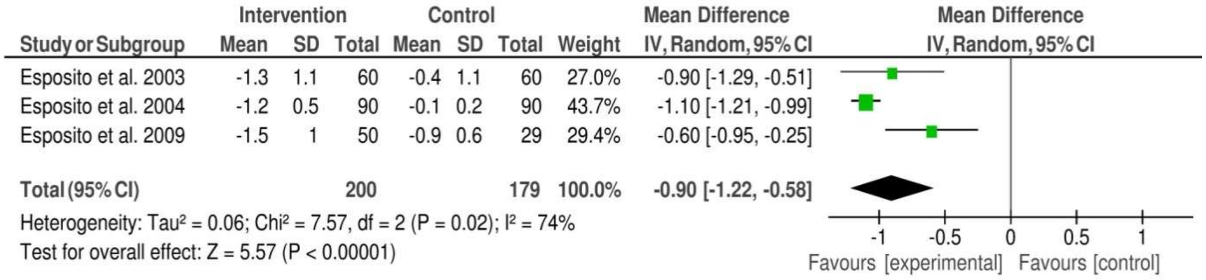

Figure S7. Forest plot for overall homeostatic model assessment of insulin resistance estimate

Mean differences were pooled using random effects meta-analysis. Squares indicate effect size for each study (mean difference between intervention and control group), with extended lines representing 95% confidence intervals (95% CIs). Diamonds indicate the overall weighted mean effect size. I² indicates between-trial heterogeneity.

All studies compared the intervention with a control condition receiving advice on both diet and PA. In one study (Esposito et al, 2003), the intervention (education on low-energy MD and advice to increase PA) was compared with general advice on healthy eating and PA [40]. In one study, the intervention was compared with a prudent diet and PA (Esposito et al, 2004: advice on a low-energy MD and ≥30 min/day of PA vs. advice on a prudent diet and ≥30 min/day of PA [39]. One study (Esposito et al, 2009) compared the intervention (low-energy, low-carbohydrate MD and ≥30 min/day of PA) to a control group receiving advice on a low-energy, low-fat diet and ≥30 min/day of PA [37].

Study or Subgroup	Intervention Mean	Intervention SD	Intervention Total	Control Mean	Control SD	Control Total	Weight	Mean Difference IV, Random, 95% CI
Esposito et al. 2003	-9	5.6	60	-2	5.6	60	39.7%	-7.00 [-9.00, -5.00]
Esposito et al. 2004	-8	3	90	-2	1.5	90	51.2%	-6.00 [-6.69, -5.31]
Esposito et al. 2009	-30.6	19.8	50	-14.4	14.4	29	9.0%	-16.20 [-23.79, -8.61]
**Total (95% CI)**			**200**			**179**	**100.0%**	**-7.32 [-9.82, -4.82]**

Heterogeneity: Tau² = 3.06; Chi² = 7.60, df = 2 (P = 0.02); I² = 74%
Test for overall effect: Z = 5.73 (P < 0.00001)

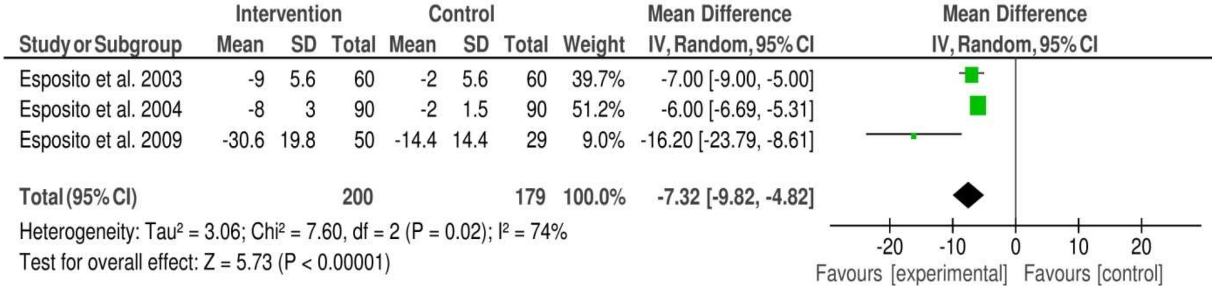

Figure S8. Forest plot for overall glucose concentrations estimate

Mean differences were pooled using random effects meta-analysis. Squares indicate effect size for each study (mean difference between intervention and control group), with extended lines representing 95% confidence intervals (95% CIs). Diamonds indicate the overall weighted mean effect size. I² indicates between-trial heterogeneity.

All studies compared the intervention with a control condition receiving advice on both diet and PA. In one study (Esposito et al, 2003), the intervention (education on low-energy MD and advice to increase PA) was compared with general advice on healthy eating and PA [40]. In one study, the intervention was compared with a prudent diet and PA (Esposito et al, 2004: advice on a low-energy MD and ≥30 min/day of PA vs. advice on a prudent diet and ≥30 min/day of PA [39]. One study (Esposito et al, 2009) compared the intervention (low-energy, low-carbohydrate MD and ≥30 min/day of PA) to a control group receiving advice on a low-energy, low-fat diet and ≥30 min/day of PA [37].

Study or Subgroup	Intervention Mean	Intervention SD	Intervention Total	Control Mean	Control SD	Control Total	Weight	Mean Difference IV, Fixed, 95% CI
Esposito et al. 2003	-19	19.6	60	-8	19.6	60	5.6%	-11.00 [-18.01, -3.99]
Esposito et al. 2004	-18	8	90	1	3	90	88.9%	-19.00 [-20.77, -17.23]
Esposito et al. 2009	-24.8	24.8	50	-6.2	8.9	29	4.8%	-18.60 [-26.20, -11.00]
Gomez-Huelgas et al. 2015	-4	83	230	6	113	176	0.7%	-10.00 [-29.84, 9.84]
**Total (95% CI)**			**430**			**355**	**100.0%**	**-18.47 [-20.13, -16.80]**

Heterogeneity: Chi² = 5.40, df = 3 (P = 0.14); I² = 44%
Test for overall effect: Z = 21.75 (P < 0.00001)

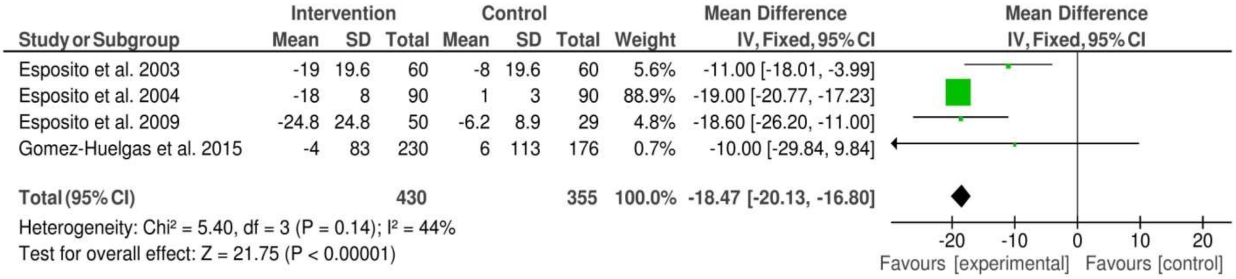

Figure S9. Forest plot for overall triglyceride concentrations estimate

Mean differences were pooled using random effects meta-analysis. Squares indicate effect size for each study (mean difference between intervention and control group), with extended lines representing 95% confidence intervals (95% CIs). Diamonds indicate the overall weighted mean effect size. I² indicates between-trial heterogeneity.

All studies compared the intervention with a control condition receiving advice on both diet and PA. In two studies, the intervention (education on low-energy MD and advice to increase PA, Esposito et al, 2003 and Gomez-Huelgas et al, 2015) was compared with general advice on healthy eating and PA [40, 41]. In one study, the intervention was compared with a prudent diet and PA (Esposito et al, 2004: advice on a low-energy MD and ≥30 min/day of PA vs. advice on a prudent diet and ≥30 min/day of PA [39]. One study (Esposito et al, 2009) compared the intervention (low-energy, low-carbohydrate MD and ≥30 min/day of PA) to a control group receiving advice on a low-energy, low-fat diet and ≥30 min/day of PA [37].

Study or Subgroup	Intervention Mean	Intervention SD	Intervention Total	Control Mean	Control SD	Control Total	Weight	Mean Difference IV, Random, 95% CI
Esposito et al. 2003	8	2.19	60	0	2.19	60	25.9%	8.00 [7.22, 8.78]
Esposito et al. 2004	4	2	90	1	1	90	26.2%	3.00 [2.54, 3.46]
Esposito et al. 2009	3.5	3.1	50	0.8	0.8	29	25.7%	2.70 [1.79, 3.61]
Gomez-Huelgas et al. 2015	4	12	230	2	12	176	22.2%	2.00 [-0.36, 4.36]
**Total (95% CI)**			**430**			**355**	**100.0%**	**3.99 [1.22, 6.77]**

Heterogeneity: Tau² = 7.57; Chi² = 128.73, df = 3 (P < 0.00001); I² = 98%
Test for overall effect: Z = 2.82 (P = 0.005)

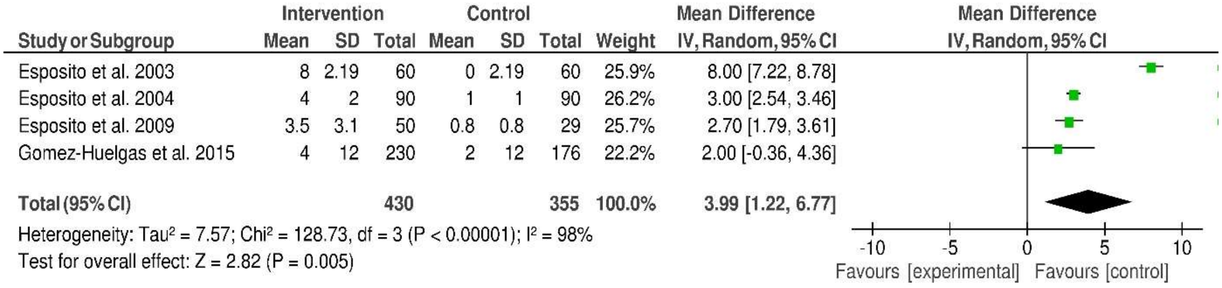

Figure S10. Forest plot for overall HDL-cholesterol concentrations estimate

Mean differences were pooled using random effects meta-analysis. Squares indicate effect size for each study (mean difference between intervention and control group), with extended lines representing 95% confidence intervals (95% CIs). Diamonds indicate the overall weighted mean effect size. I² indicates between-trial heterogeneity.

All studies compared the intervention with a control condition receiving advice on both diet and PA. In two studies, the intervention (education on low-energy MD and advice to increase PA, Esposito et al, 2003 and Gomez-Huelgas et al, 2015) was compared with general advice on healthy eating and PA [40, 41]. In one study, the intervention was compared with a prudent diet and PA (Esposito et al, 2004: advice on a low-energy MD and ≥30 min/day of PA vs. advice on a prudent diet and ≥30 min/day of PA [39]. One study (Esposito et al, 2009) compared the intervention (low-energy, low-carbohydrate MD and ≥30 min/day of PA) to a control group receiving advice on a low-energy, low-fat diet and ≥30 min/day of PA [37].

Study or Subgroup	Intervention Mean	Intervention SD	Intervention Total	Control Mean	Control SD	Control Total	Weight	Mean Difference IV, Random, 95% CI
Esposito et al. 2003	-4	22.3	60	0	22.3	60	12.4%	-4.00 [-11.98, 3.98]
Esposito et al. 2004	-11	6	90	-2	2	90	42.9%	-9.00 [-10.31, -7.69]
Esposito et al. 2009	-9.7	7.7	50	-3.9	7	29	31.3%	-5.80 [-9.12, -2.48]
Gomez-Huelgas et al. 2015	-9	40	230	-8	37	176	13.5%	-1.00 [-8.52, 6.52]
**Total (95% CI)**			**430**			**355**	**100.0%**	**-6.30 [-9.59, -3.02]**

Heterogeneity: $Tau^2 = 6.10$; $Chi^2 = 8.01$, df = 3 (P = 0.05); $I^2 = 63\%$
Test for overall effect: Z = 3.76 (P = 0.0002)

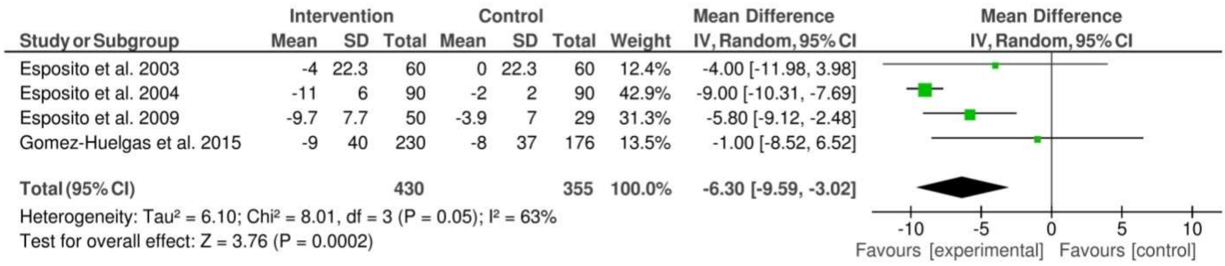

Figure S11. Forest plot for overall total cholesterol concentrations estimate

Mean differences were pooled using random effects meta-analysis. Squares indicate effect size for each study (mean difference between intervention and control group), with extended lines representing 95% confidence intervals (95% CIs). Diamonds indicate the overall weighted mean effect size. I^2 indicates between-trial heterogeneity.

All studies compared the intervention with a control condition receiving advice on both diet and PA. In two studies, the intervention (education on low-energy MD and advice to increase PA, Esposito et al, 2003 and Gomez-Huelgas et al, 2015) was compared with general advice on healthy eating and PA [40, 41]. In one study, the intervention was compared with a prudent diet and PA (Esposito et al, 2004: advice on a low-energy MD and ≥30 min/day of PA vs. advice on a prudent diet and ≥30 min/day of PA [39]. One study (Esposito et al, 2009) compared the intervention (low-energy, low-carbohydrate MD and ≥30 min/day of PA) to a control group receiving advice on a low-energy, low-fat diet and ≥30 min/day of PA [37].

Supplementary Materials I, Figure S12. Proportion of included articles reporting each of the CONSORT checklist items

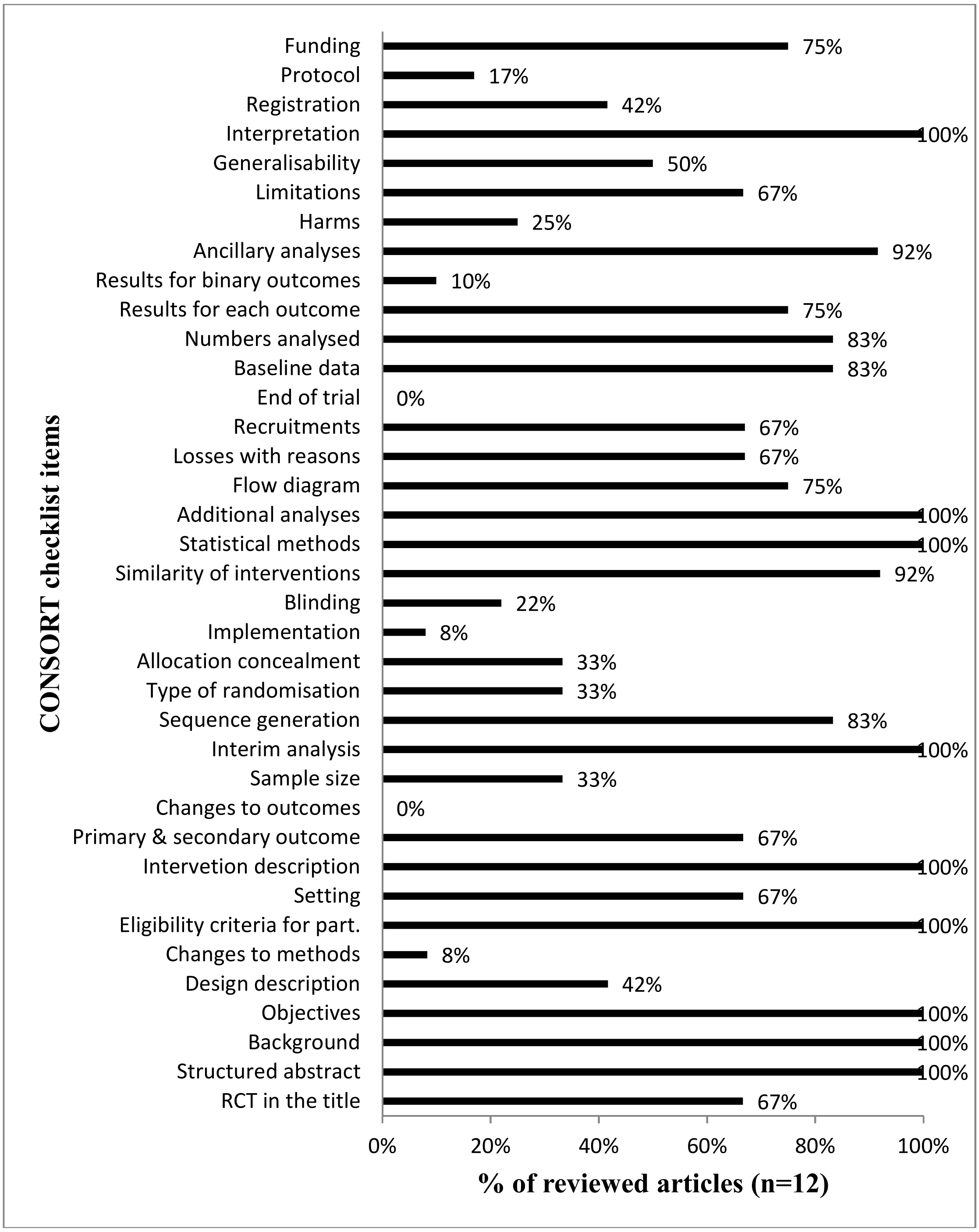

Supplementary Materials I, Figure S13. Detailed risk of bias for each included article

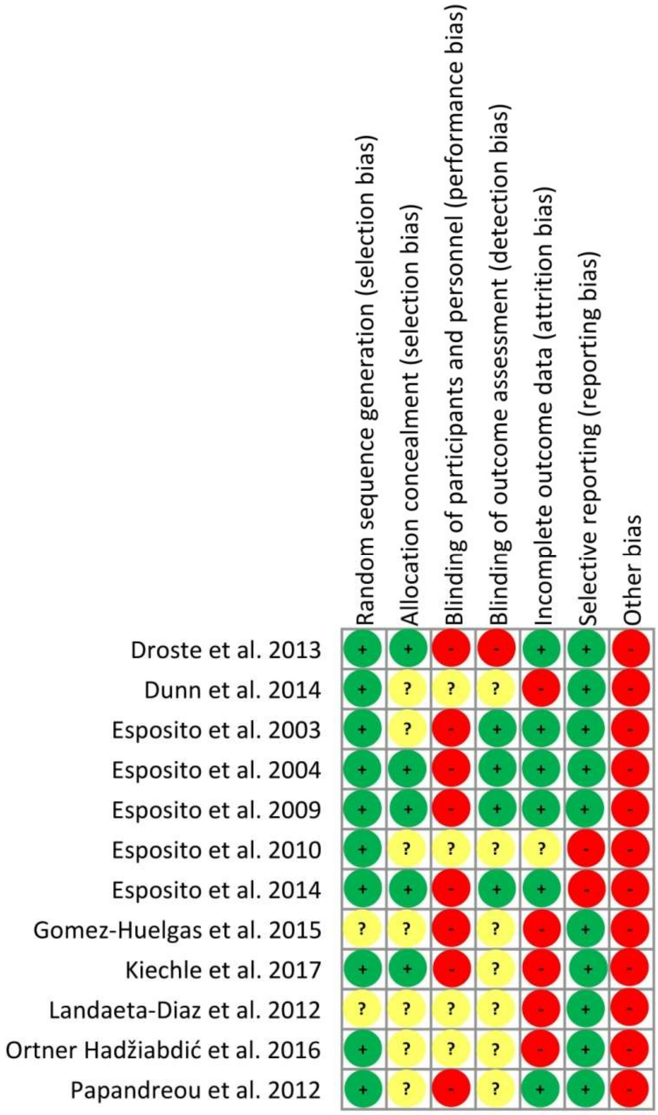

Supplementary Materials I. Funnel plots of randomised controlled trials evaluating the combined effect of the Mediterranean diet and physical activity on metabolic risk factors (SE, standard error; MD, mean difference)

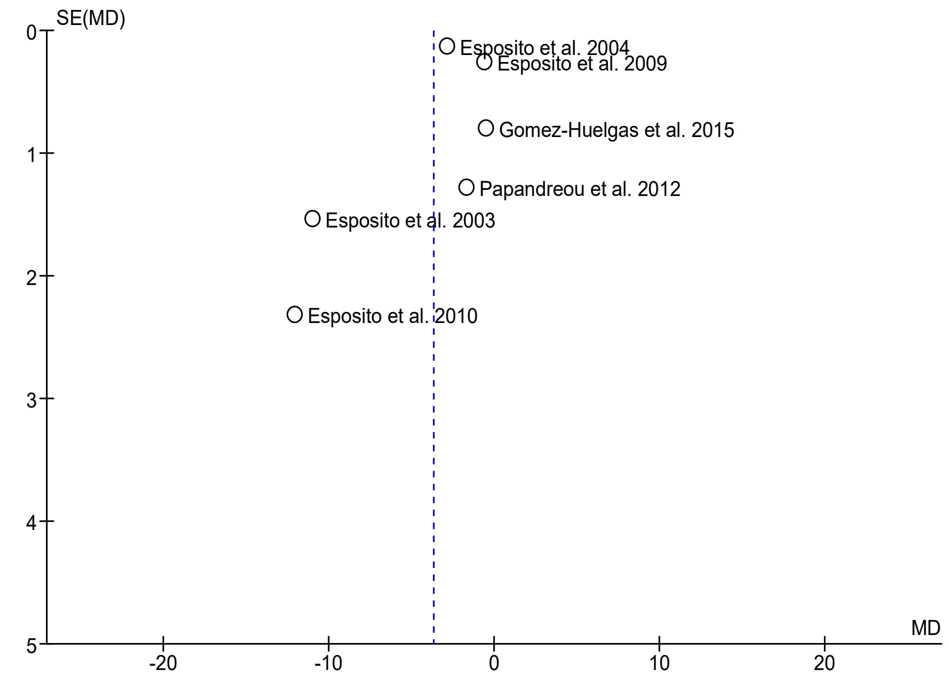

Figure S14. Funnel plot of combined effect of the Mediterranean diet and physical activity on body weight

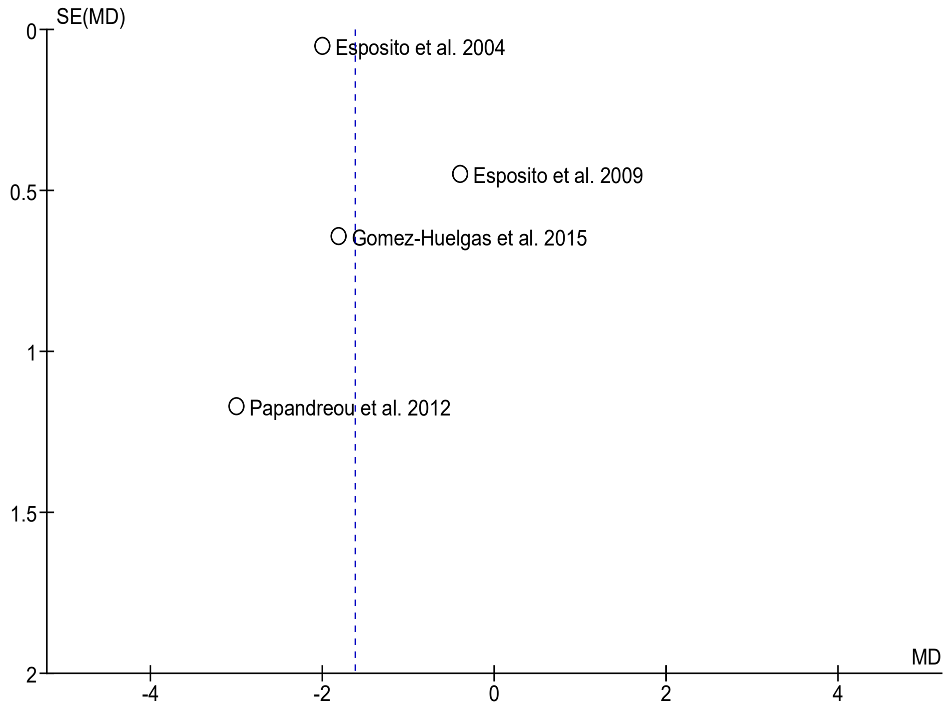

Figure S15. Funnel plot of combined effect of the Mediterranean diet and physical activity on WC.

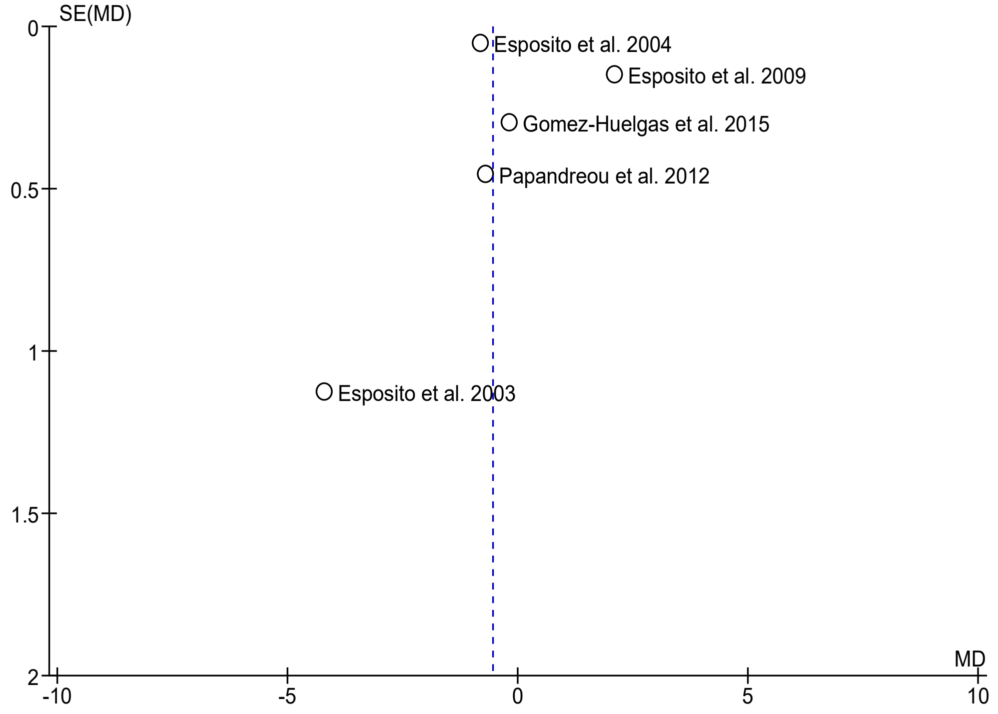

Figure S16. Funnel plot of combined effect of the Mediterranean diet and physical activity on BMI

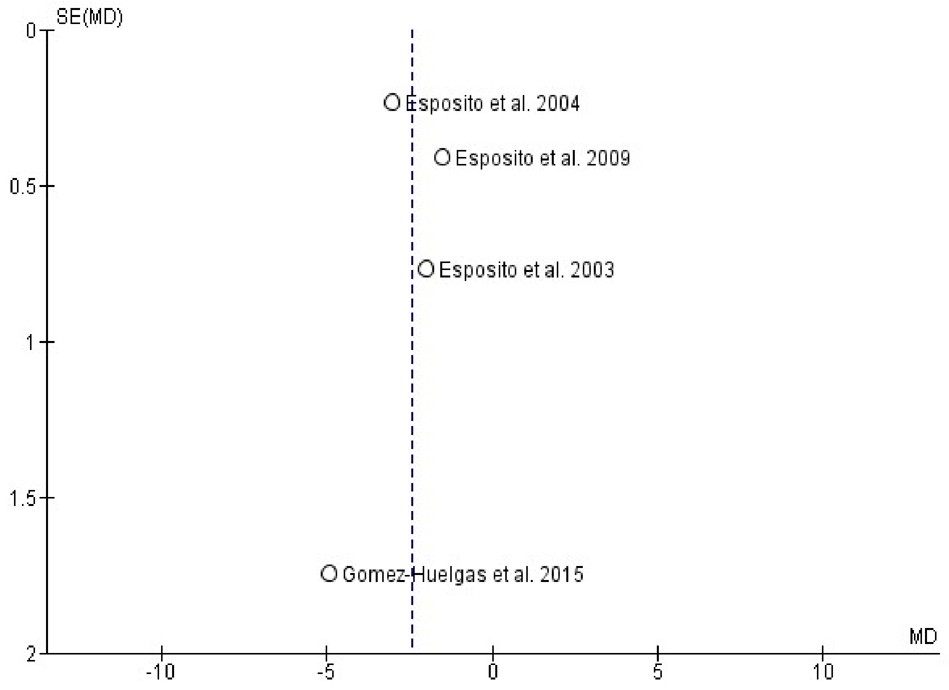

Figure S17. Funnel plot of combined effect of the Mediterranean diet and physical activity on SBP

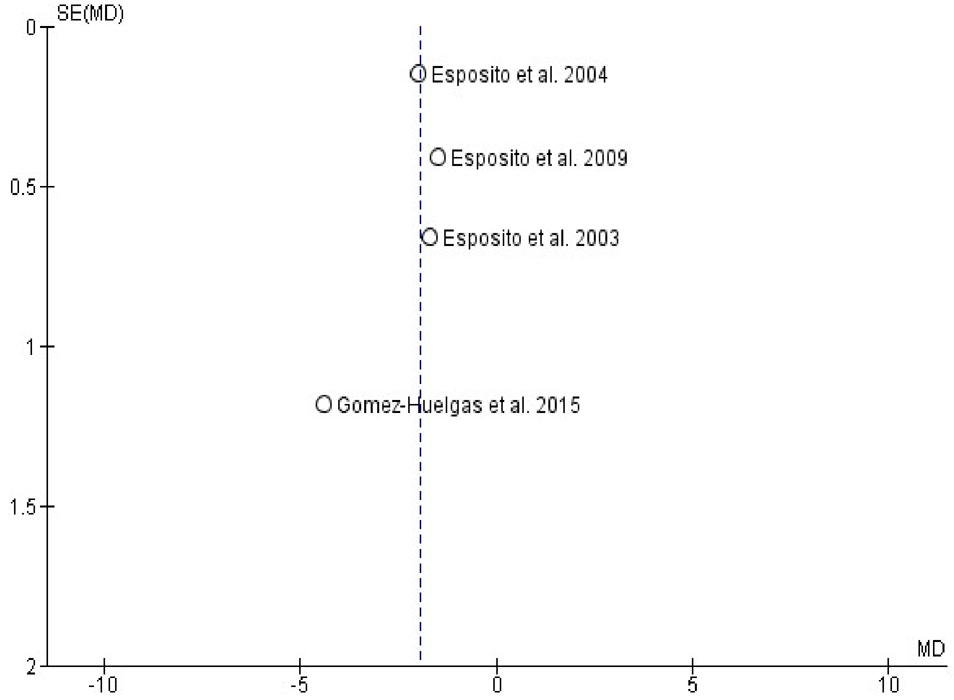

Figure S18. Funnel plot of combined effect of the Mediterranean diet and physical activity on DBP

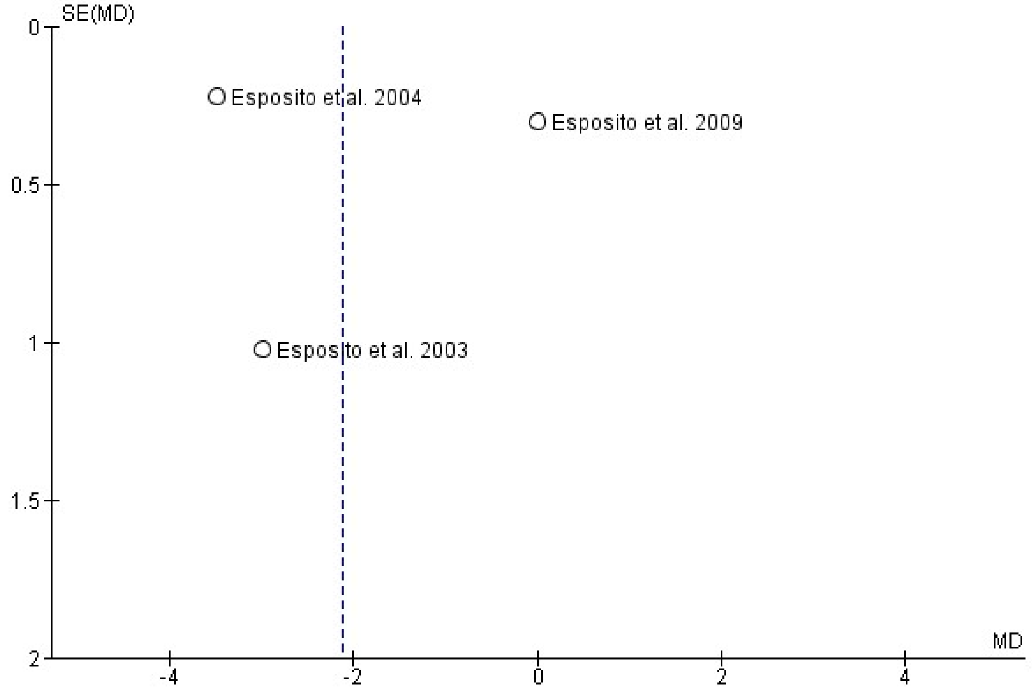

Figure S19. Funnel plot of combined effect of the Mediterranean diet and physical activity on insulin concentrations

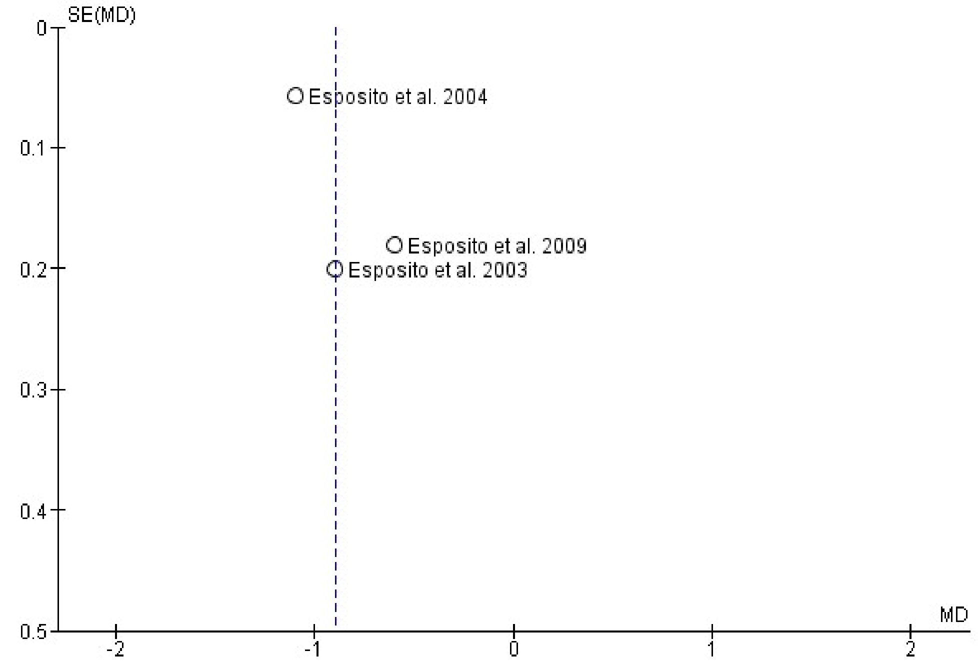

Figure S20. Funnel plot of combined effect of the Mediterranean diet and physical activity on HOMA-IR

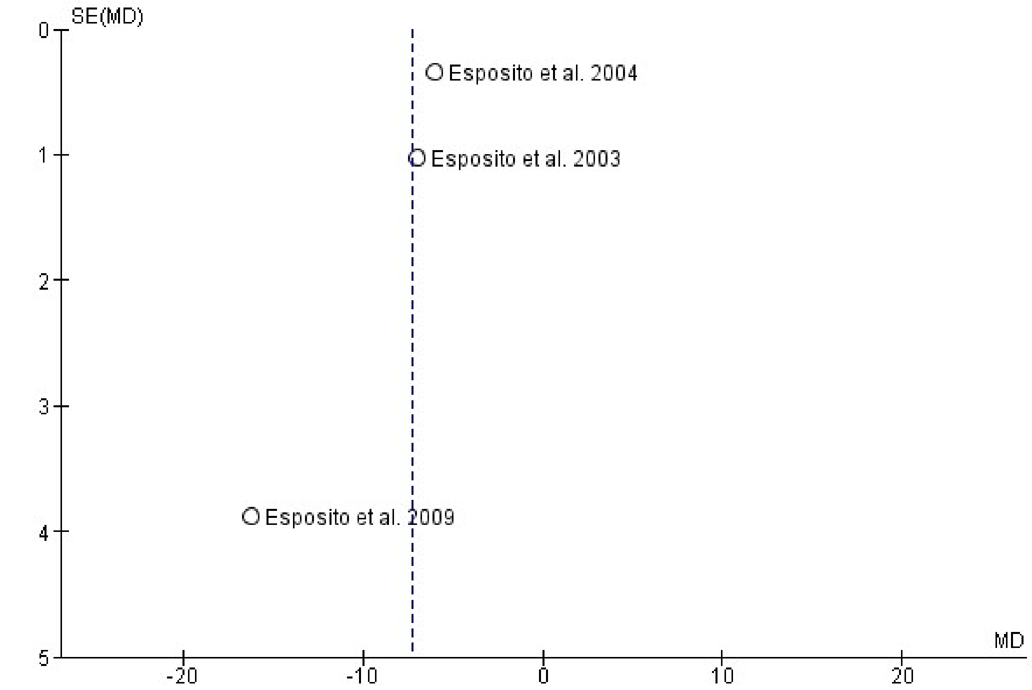

Figure S21. Funnel plot of combined effect of the Mediterranean diet and physical activity on blood glucose concentrations

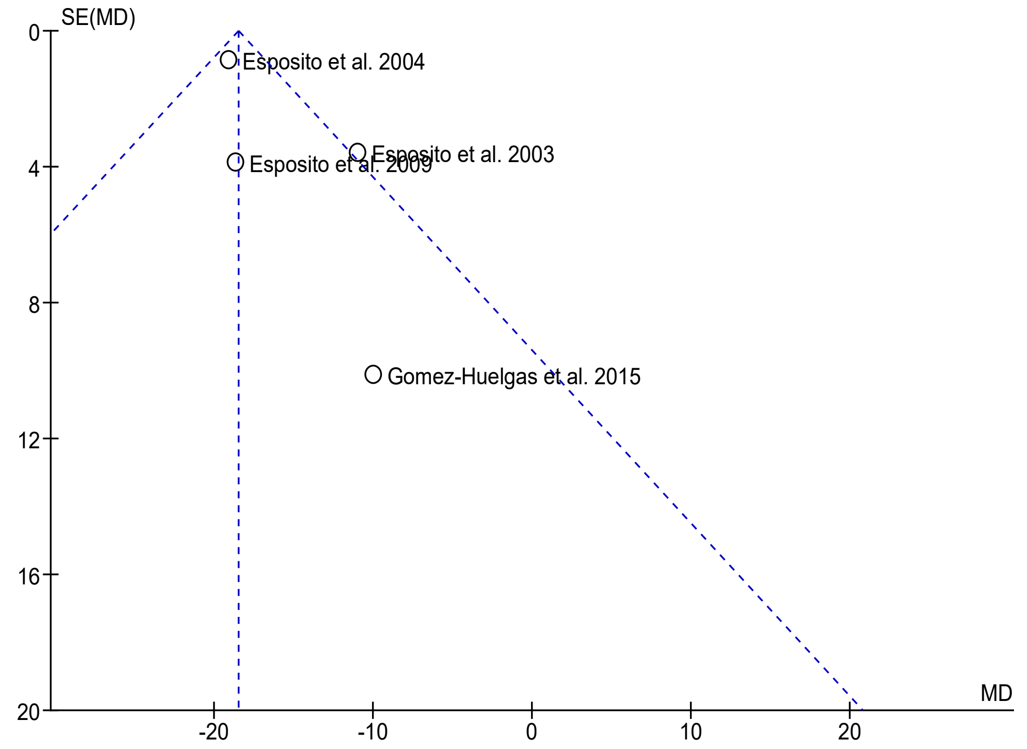

Figure S22. Funnel plot of combined effect of the Mediterranean diet and physical activity on triglyceride concentrations

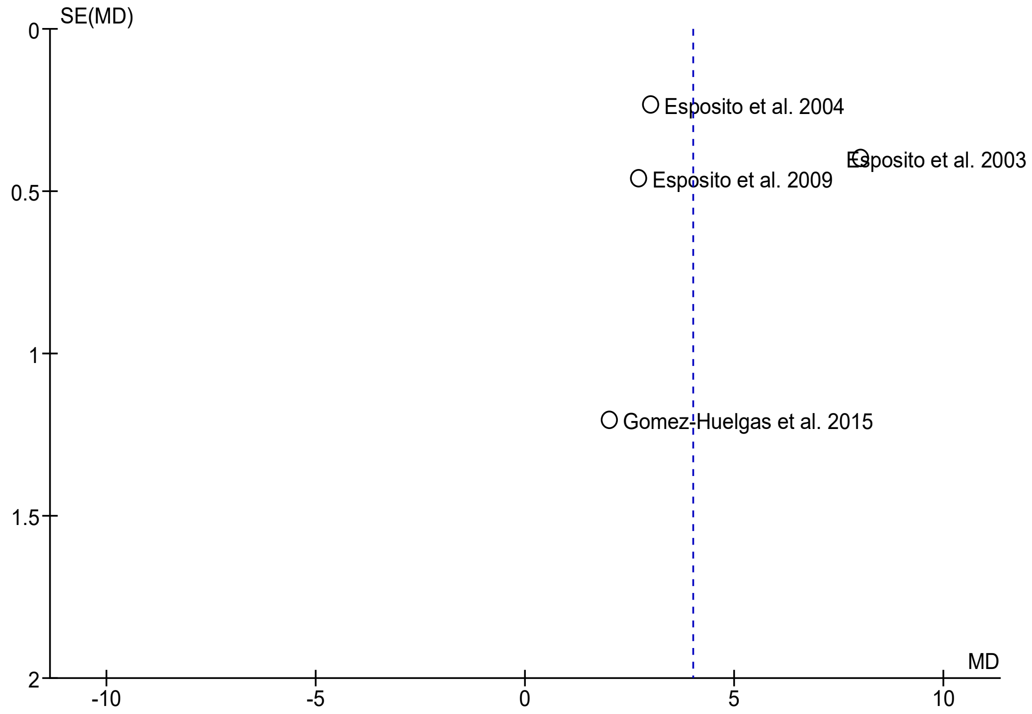

Figure S23. Funnel plot of combined effect of the Mediterranean diet and physical activity on HDL-cholesterol concentrations

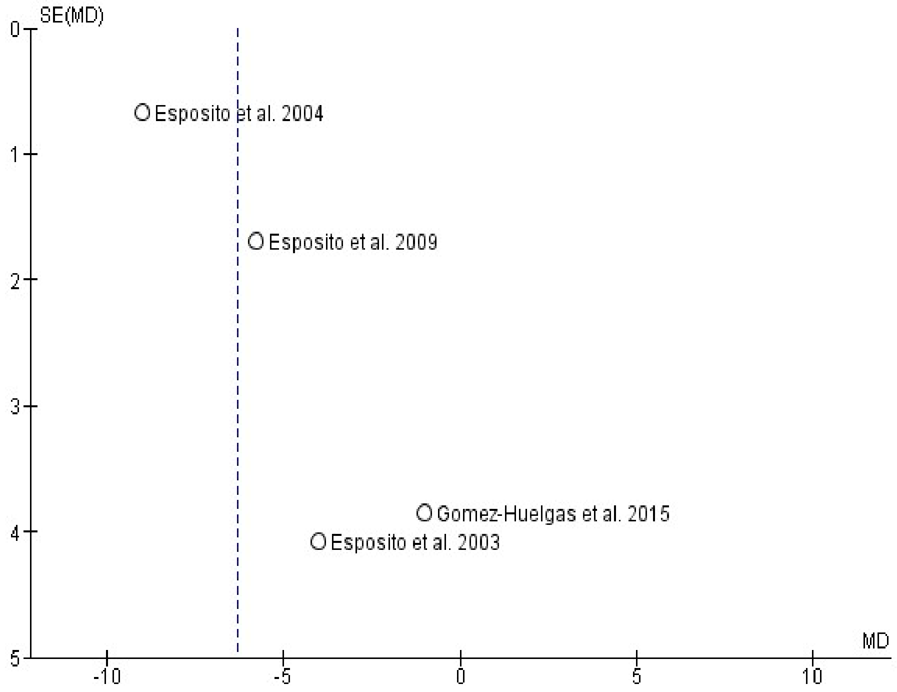

Figure S24. Funnel plot of combined effect of the Mediterranean diet and physical activity on total cholesterol concentrations
