# Supplementary material for: The Combined Effect of Promoting the Mediterranean Diet and Physical Activity on Metabolic Risk Factors in Adults: A Systematic Review and Meta-Analysis of Randomised Controlled Trials
Source: Nutrients. 2018 Oct 25;10(11):1577. doi: 10.3390/nu10111577 (PMC6267322; doi:10.3390/nu10111577)
Supplement: Supplementary file 1 [file nutrients-10-01577-s001.zip › nutrients-372737-supplementary/nutrients-372737-proof done-supplementaty II.pdf]

Supplementary Materials II. Search strategy

Databases searched until 27th March 2018.

Strategy for Medline

1."mediterranean diet" or "mediterranean lifestyle" or "meddiet score" or "meddiet" or "mediterranean style diet" or "mediterranean diet score" or "mediterranean diet index"

2. Diet, Mediterranean [Mesh]

3. Exercise or exercises or training or "physical activity" or "physical activities" or "moderate physical activity" or "vigorous physical activity" or "strenuous physical activity" or sport or sports or accelerometer or accelerometers or pedometer or pedometers or walk or walking or run or running or jog or jogging

4. Exercise [Mesh]

5 "metabolic risks" or "metabolic risk" or "metabolic markers" or "metabolic syndrome" or "cardiovascular disease" or CVD or "cardiovascular risk factors" or "cardiovascular disease risk" or "cardiovascular disease risks" or "vascular markers" or adiposity or overweight or obesity or obese or "body weight" or "body composition" or BMI or "body mass" or "waist circumference" or "weight loss" or weight or "blood pressure" or cholesterol or triglycerides

6. Obesity [Mesh]

7. Cardiovascular diseases [Mesh]

8. Intervention or interventions or "controlled trial" or "controlled trials" or rct or rcts or "randomized controlled trial" or "randomised controlled trial"

9. #1 or #2

10. #3 or #4

11. #5 or #6 or #7

Final search: #9 AND #10 AND #11 AND #8 (No limitations were used)

Results: 249

Strategy for Embase

1."mediterranean diet" or "mediterranean lifestyle" or "meddiet score" or "meddiet" or "mediterranean style diet" or "mediterranean diet score" or "mediterranean diet index"

2. Exercise or exercises or training or "physical activity" or "physical activities" or "moderate physical activity" or "vigorous physical activity" or "strenuous physical activity" or sport or sports or accelerometer or accelerometers or pedometer or pedometers or walk or walking or run or running or jog or jogging

3. "metabolic risks" or "metabolic risk" or "metabolic markers" or "metabolic syndrome" or "cardiovascular disease" or CVD or "cardiovascular risk factors" or "cardiovascular disease risk" or

"cardiovascular disease risks" or "vascular markers" or adiposity or overweight or obesity or obese or "body weight" or "body composition" or BMI or "body mass" or "waist circumference" or "weight loss" or weight or "blood pressure" or cholesterol or triglycerides

4. Intervention or interventions or "controlled trial" or "controlled trials" or rct or rcts or "randomized controlled trial" or "randomised controlled trial"

Final search: #1 AND #2 AND #3 AND #4 (No limitations were used)

Results: 649

Strategy for Web of Science

Final search: "mediterranean diet*" or "mediterranean lifestyle*" or "mediterranean dietary pattern" or "mediterranean style diet*" or "MedDiet score" or "mediterranean diet score" or "mediterranean diet index" or MedDiet) AND "physical activit*" or "physical exercis*" or exercis* or walk* or jog* or run* or "moderate activit*" or "vigorous activit*" or accelomet* or pedomet* or sport* AND "metabolic risk*" or "metabolic marker*" or "metabolic syndrome" or "cardiovascular disease*" or CVD or "vascular marker*" or "blood pressure" or cholesterol or triglycerides or adiposity or overweight or obese or obesity or "body weight" or "body composition" or BMI or "body mass" or "waist circumference" or weight or "weight loss" AND intervention* or "controlled trial*" or "randomized controlled trial*" or rct or rcts (No limitations were used)

Results: 375

Strategy for CINAHL

1. "mediterranean diet" or "mediterranean lifestyle" or "meddiet score" or "meddiet" or "mediterranean style diet" or "mediterranean diet score" or "mediterranean diet index"
2. Exercise or exercises or training or "physical activity" or "physical activities" or "moderate physical activity" or "vigorous physical activity" or "strenuous physical activity" or sport or sports or accelerometer or accelerometers or pedometer or pedometers or walk or walking or run or running or jog or jogging
3. "metabolic risks" or "metabolic risk" or "metabolic markers" or "metabolic syndrome" or "cardiovascular disease" or CVD or "cardiovascular risk factors" or "cardiovascular disease risk" or "cardiovascular disease risks" or "vascular markers" or adiposity or overweight or obesity or obese or "body weight" or "body composition" or BMI or "body mass" or "waist circumference" or "weight loss" or weight or "blood pressure" or cholesterol or triglycerides
4. intervention or interventions or "controlled trial" or "controlled trials" or rct or rcts or "randomized controlled trial" or "randomised controlled trial"

Final search: #1 AND #2 AND #3 AND #4 (No limitations were used)

Results: 69
